# Nitrogen-Stabilized Fe–Co Nanoclusters Supported on Mushroom-Residue-Derived Porous Carbon for Peroxymonosulfate Activation Toward Sulfadiazine Degradation

**DOI:** 10.3390/molecules31193392

**Published:** 2026-09-24

**Authors:** Tianlong Zhang, Yuxin Guo, Xiqi Xu, Changqing Hu, Bei Chu

**Affiliations:** Ningbo Key Laboratory of Agricultural Germplasm Resources Mining and Environmental Regulation, College of Science and Technology, Ningbo University, Cixi 315300, China; zhangtianlong2026@126.com (T.Z.);

**Keywords:** sulfadiazine, peroxymonosulfate, Fe–Co nanoclusters, biomass-derived porous carbon, radical oxidation

## Abstract

Addressing the environmental persistence and ecotoxicological risks of the antibiotic sulfadiazine (SDZ) in water bodies is a pressing concern. In this study, nitrogen-stabilized Fe–Co nanoclusters supported on mushroom-residue-derived N-doped porous carbon (FeCo/NPC-800) were fabricated via a dicyandiamide-assisted pyrolysis strategy and used to activate peroxymonosulfate (PMS) for SDZ degradation. Synchrotron radiation X-ray absorption fine structure measurements and quantitative fitting indicated the coexistence of metal–heteroatom and metal–metal coordination, consistent with ultrasmall, structurally disordered Fe/Co-containing domains anchored to the N-doped carbon matrix. The FeCo/NPC-800/PMS system achieved an SDZ removal efficiency of 91.2% within 60 min, confirming that FeCo/NPC-800 can effectively activate PMS under the tested conditions. Radical-quenching experiments and electron spin resonance analysis identified sulfate and hydroxyl radicals, with sulfate radicals appearing to make the larger contribution under the tested conditions. Degradation intermediates collected at 10 and 40 min were analyzed by liquid chromatography–mass spectrometry, and three main transformation pathways were proposed: amino oxidation followed by sulfur dioxide extrusion, sulfur–nitrogen bond cleavage, and pyrimidine-ring opening. These findings provide insights into biomass-derived, nitrogen-stabilized bimetallic carbon catalysts for PMS-based treatment of refractory antibiotic contaminants.

## 1. Introduction

Currently, the extensive use of pharmaceuticals and personal care products (PPCPs) is a major cause of water pollution [1]. Antibiotics are among the most extensively studied PPCPs [2,3] owing to their essential role in protecting human health [4]. However, antibiotic misuse leads to persistent residues in production wastewater, posing serious threats to the ecological environment [5]. In particular, sulfadiazine (SDZ) is widely used in animal husbandry and human medicine because of its low cost, broad spectrum, and high stability [6]. SDZ exhibits inherent biological activity and is resistant to degradation in soil, aqueous environments, and living organisms [7]. Its persistence can adversely affect the functional and structural diversity of environmental microbial communities [8]. Through biological and chemical processes, SDZ residues may promote the generation and enrichment of corresponding antibiotic-resistance genes (e.g., sul), undergo various transformations, and yield multiple metabolites [9]. Therefore, investigating SDZ degradation technologies and elucidating their mechanisms are important for addressing contamination in water and soil.

Advanced oxidation processes (AOPs), including photolysis [10], Fenton oxidation [11], and ozonation [12], have been investigated for SDZ treatment; however, individual processes may show limited efficiency or require relatively demanding operating conditions. Sulfate-radical-based AOPs using peroxymonosulfate (PMS) are attractive because appropriate activation can generate reactive oxygen species such as sulfate radicals (SO_4_•^−^) and hydroxyl radicals (•OH), enabling rapid oxidation of a wide range of organic pollutants [13,14,15]. Heterogeneous carbon-based PMS activators are attractive because of their relatively low cost, convenient recovery, chemical stability, and tunable surface properties [16,17]. Nevertheless, the limited density of accessible active sites and inefficient interfacial electron transfer in unmodified carbon materials can restrict their catalytic performance [18]. Biomass-derived porous carbons provide a sustainable alternative because their pore structure, surface functionality, and carbon framework can be adjusted through activation and heteroatom doping. Nitrogen-doped biochar and N-coordinated metal-containing porous carbon have accordingly shown considerable potential for PMS activation [19,20].

Heteroatom doping is an effective strategy for regulating the electronic, chemical, and structural properties of carbon catalysts. Nitrogen, oxygen, sulfur, and boron dopants can modify charge distribution, create defects, and introduce new adsorption or reaction sites [21,22]. The combination of heteroatom doping with a hierarchical pore structure can further improve active-site dispersion and accessibility [23], while nitrogen configurations in the carbon framework may promote PMS adsorption and interfacial electron transfer [24]. In particular, N-doped porous carbon provides abundant coordination environments for stabilizing transition-metal species and suppressing their aggregation, thereby coupling porous mass transfer with metal–nitrogen active centers [25].

Transition-metal modification can further enhance the ability of biochar and porous carbon to activate PMS. Bimetallic catalysts are especially attractive because adjacent metal centers can provide complementary adsorption and redox functions, as demonstrated for transition-metal-loaded biochar systems [26]. Fe- and Co-containing carbon materials have also been widely investigated because high-temperature treatment can promote carbon-framework reconstruction and because the metal centers participate in interfacial redox processes [27,28]. Compared with a single-metal catalyst, a bimetallic system can alleviate limitations associated with a single catalytic function and may improve the dispersion and stability of active species [29,30]. Electronic coupling between two metals can redistribute charge and facilitate valence-state cycling and electron transfer [31,32]. Iron-containing catalysts can activate PMS through redox-active Fe centers and iron–heteroatom sites [33]. Incorporating Fe into nitrogen-containing carbon can also regulate the electronic structure and generate defect-associated adsorption and catalytic sites [34,35,36]. When coupled with cobalt, Fe can induce intermetallic charge redistribution, whereas cobalt contributes additional redox-active centers [37]. Despite these advantages, the coordination structure and PMS-activation behavior of ultrasmall Fe–Co nanoclusters supported on biomass-derived N-doped porous carbon remain insufficiently understood.

Biochar is a carbon-rich material obtained by biomass pyrolysis and can provide a porous framework, oxygen-containing surface groups, and adsorption sites [38]. Although pristine biochar generally exhibits limited PMS-activation ability, metal incorporation can markedly improve interfacial electron transfer and catalytic reactivity [39]. In a bimetallic biochar catalyst, the different redox characteristics of the two metals may facilitate valence cycling and electron migration, while the carbon matrix helps disperse and stabilize the active species. However, many high-performance metal–carbon catalysts rely on refined commercial carbon supports or multistep synthesis, increasing material and processing costs. Converting mushroom residue into a porous carbon support offers a resource-recovery route for this biomass waste. Previous work has shown that Agaricus bisporus residue can be transformed into hierarchically porous Fe/N co-doped carbon for the oxygen reduction reaction [40]. Nitrogen incorporation can provide coordination sites for anchoring and stabilizing metal species within the carbon framework [41].

In this study, mushroom-residue-derived activated carbon (MRB) is used to prepare N-doped porous carbon supporting small Fe/Co-containing domains through dicyandiamide-assisted pyrolysis. The resulting catalysts are designated FeCo/NPC-T, where NPC denotes nitrogen-doped porous carbon and T represents the pyrolysis temperature. Compared with the previously reported mushroom-residue-derived Fe/N carbon, the present work introduces a bimetallic Fe–Co system for PMS activation and sulfadiazine degradation, with particular attention to local metal coordination and the contribution of incorporating both metals. Synchrotron-based XAFS is used to characterize metal–N/O coordination and metal–metal contacts within the supported domains. Comparisons with the metal-free support and corresponding monometallic catalysts assess the catalytic performance of the bimetallic material under identical reaction conditions. The relationship between pyrolysis temperature, pore structure, and degradation kinetics is examined to explain the selection of the preparation temperature. The effects of reaction conditions on SDZ removal are evaluated, together with catalyst reusability and metal leaching. Radical-quenching experiments and ESR spectroscopy are used to investigate the reactive species involved in PMS activation, while HPLC–MS analysis identifies transformation products for the proposed SDZ degradation pathways.

## 2. Results and Discussion

### 2.1. Characterization of the Prepared Catalysts

The morphologies of pristine MRB and FeCo/NPC-800 were characterized by SEM. As shown in Figure 1a–c, MRB contains irregular carbonaceous fragments, lamellar features, and open pore-like voids over multiple length scales. At higher magnification, the surface remains rough and heterogeneous. Compared with MRB, FeCo/NPC-800 exhibits a more fragmented porous surface composed of irregular lamellar carbon domains and fine carbonaceous particles (Figure 1d–f). Abundant folds, voids, and interconnected pore-like features indicate reconstruction of the carbon matrix during dicyandiamide-assisted pyrolysis.

TEM images reveal wrinkled and locally stacked carbon lamellae with irregular outlines (Figure 2a–f). At higher magnification, locally ordered fringes are observed within the predominantly disordered carbon matrix, indicating the coexistence of short-range ordered regions and an amorphous carbon structure.

Figure 3 compares the XPS spectra of fresh FeCo/NPC-800 (Figure 3a–e) and the catalyst after the standard 60 min reaction (Figure 3f–j); the survey spectra are provided in Figure S1. The C 1s spectra display a dominant component near 284.0 eV characteristic of sp^2^ carbon. Components at approximately 284.8, 286.1, 287.7, and 289.9 eV are tentatively assigned to C–C/C–H, C–O/C–N, C=O, and higher-binding-energy π–π*/O–C=O contributions, respectively [42]. The N 1s components are assigned to C=N, C–N, and N–O environments [43], and the O 1s components are assigned to metal–oxygen-related species, C=O, and C–O groups [44]. The Co 2p envelopes contain overlapping Co(II), Co(III), and shake-up-satellite contributions, whereas the Fe 2p components near 709.3 and 710.8 eV are assigned to Fe(II) and Fe(III), respectively [45,46]. Detailed peak positions, FWHM values, background treatment, and handling of the satellite components are provided in Tables S4 and S5. The fitted Co(III) proportion increased from 21.87% in the fresh catalyst to 28.69% after reaction, whereas the fitted Fe oxidation-state distribution changed only slightly. These ex situ differences indicate surface oxidation-state redistribution following PMS exposure and are consistent with participation of metal-containing sites in PMS activation. Based on these XPS changes, the proposed involvement of Fe/Co-containing surface sites in PMS activation is summarized in Scheme 1.

Figure 4a presents the XRD patterns of MRB and FeCo/NPC-800. MRB exhibits a broad diffraction feature near 26.8°, corresponding to the (002) reflection of turbostratic or graphitic carbon and indicating a predominantly disordered carbon structure [47,48]. FeCo/NPC-800 exhibits diffraction features at approximately 26.8° and 45.1°. The feature at 45.1° reflects contributions from the (100) plane of locally ordered graphitic carbon and the (110) plane of bcc FeCo, indicating the formation of locally ordered carbon and FeCo-containing domains after Fe/Co incorporation [49,50]. Raman spectroscopy (Figure 4b) shows characteristic D and G bands at approximately 1386.42 and 1587.74 cm^−1^, respectively. The D-to-G band intensity ratio of 0.70 indicates that the carbon framework contains both defect-associated sites and ordered sp^2^-carbon domains [51]. The FTIR spectra (Figure 4c) exhibit vibrations associated with O–H, C=O, aromatic C=C/C–H, and C–O groups. The band near 635 cm^−1^ is assigned to Fe–O vibration, indicating the presence of Fe–O bonding in FeCo/NPC-800 [52].

To clarify the local coordination environment of the metal sites in FeCo/NPC-800, Co K-edge and Fe K-edge XAFS spectra were analyzed [53,54]. The XANES edge positions and spectral profiles (Figure 5a,d) differ from those of the corresponding metal foils, indicating partially oxidized average electronic states of Fe and Co associated with their coordination to the surrounding heteroatoms and carbon matrix. The Fourier-transformed EXAFS spectra (Figure 5b,e) exhibit dominant first-shell features at R + α ≈ 1.8–2.2 Å, corresponding to metal–N/O coordination. The weaker features at R + α ≈ 2.4–2.6 Å are assigned to metal scatterers, indicating the presence of metal–metal coordination. Wavelet-transform analysis (Figure 5c,f) further distinguishes the lower-k signals associated with light-atom scatterers from the higher-k signals associated with metal scatterers. Quantitative fitting (Tables S2 and S3) identifies two M–N coordination contributions with fitted coordination numbers of approximately 2.0 and 2.3, respectively, together with M–M coordination. The corresponding k-space oscillations and fitted curves are shown in Figures S4 and S5, respectively. The attenuated higher-shell features relative to the metal foil and oxide references demonstrate limited long-range structural ordering. Taken together, the XAFS results reveal a mixed local structure comprising metal–nitrogen coordination and metal–metal contacts within structurally disordered Fe/Co-containing domains dispersed on the N-doped carbon matrix.

As shown in Figure S2 and Table S1, FeCo/NPC-800 exhibits a type IV nitrogen adsorption–desorption isotherm and a hierarchical pore structure containing micropores and mesopores. Its BET surface area, total pore volume, and micropore volume are 423.5 m^2^ g^−1^, 0.3403 cm^3^ g^−1^, and 0.1522 cm^3^ g^−1^, respectively. Although FeCo/NPC-900 has a substantially larger surface area, the kinetic comparison below shows that FeCo/NPC-800 has the larger apparent rate constant over 2–30 min; thus, surface area alone does not determine PMS-activation performance. Accessible Fe/Co-containing sites, local coordination, carbon ordering, and pore-network transport may also contribute.

ICP-OES analysis of FeCo/NPC-800 gave Fe, Co, and residual Zn contents of 20.638 ± 0.042, 25.449 ± 0.031, and 0.0152 ± 0.0001 wt%, respectively (mean ± SD of triplicate instrumental readings; Table S6). The nominal Fe:Co precursor ratio was 1:1, whereas the measured Fe:Co atomic ratio in the final catalyst was 0.856:1. Treating the non-Fe/Co/Zn balance operationally as the supporting matrix gives an approximate (Fe + Co):matrix mass ratio of 0.855:1. The trace Zn content indicates that most ZnCl_2_ used for activation was removed during washing.

### 2.2. PMS Activation by FeCo/NPC-800 for SDZ Degradation

Figure 6a separates spontaneous PMS oxidation, adsorption by FeCo/NPC-800, and the combined FeCo/NPC-800/PMS process. After 60 min, PMS alone produced only 15.4% SDZ removal, whereas FeCo/NPC-800 alone removed part of the aqueous SDZ through adsorption and surface interactions. The combined FeCo/NPC-800/PMS system achieved 91.2% removal, demonstrating that FeCo/NPC-800 effectively activates PMS and accelerates the oxidative transformation of SDZ. Apparent pseudo-first-order fitting over 2–30 min gave rate constants of 0.0016, 0.0142, and 0.06060 min^−1^ for PMS, FeCo/NPC-800, and FeCo/NPC-800/PMS, respectively (Table S7). The corresponding apparent pseudo-first-order fitting plots for the systems shown in Figure 6 are provided in Figure S7.

The catalyst-preparation temperature also affected SDZ removal (Figure 6b). The 60 min removal efficiencies for FeCo/NPC-700, FeCo/NPC-800, and FeCo/NPC-900 were 67.3%, 91.2%, and 93.5%, respectively. Because the endpoint values for the 800 and 900 °C samples were close, their catalytic performance was further compared using apparent kinetic constants. Over 2–30 min, the apparent rate constants were 0.03034, 0.06060, and 0.05438 min^−1^ for the 700, 800, and 900 °C samples, respectively (R^2^ = 0.99, 0.98, and 0.99; Table S7). FeCo/NPC-800 exhibited the highest apparent rate constant and was therefore selected for the subsequent experiments. The structure–performance comparison in Table S9 shows that the larger BET surface area of FeCo/NPC-900 did not produce a higher apparent rate constant. Higher pyrolysis temperatures can alter nitrogen content and configuration as well as metal dispersion, potentially offsetting the benefit of increased surface area for PMS activation [55]. These changes could reduce the population of accessible, reactive Fe/Co-containing sites in FeCo/NPC-900. Thus, increasing the pyrolysis temperature improved the measured surface area but did not further accelerate SDZ removal over 2–30 min. FeCo/NPC-800 was selected on this kinetic basis, while FeCo/NPC-900 gave a slightly higher removal efficiency at 60 min.

To evaluate the contributions of the N-doped carbon support and metal incorporation, NPC-800, Fe/NPC-800, Co/NPC-800, and FeCo/NPC-800 were compared under identical PMS-activation conditions (Figure 6c). NPC-800/PMS exhibited limited SDZ removal, whereas both Fe/NPC-800 and Co/NPC-800 showed enhanced PMS-activation performance. FeCo/NPC-800 achieved the highest SDZ removal among the four catalysts. These results demonstrate that the simultaneous incorporation of Fe and Co provides a clear catalytic-performance advantage over the N-doped carbon support and the corresponding monometallic catalysts, supporting the cooperative participation of Fe- and Co-containing sites in PMS activation.

The effects of catalyst loading, initial pH, PMS concentration, and initial SDZ concentration are shown in Figure 7. Increasing the FeCo/NPC-800 loading from 0.2 to 0.8 and 1.0 g L^−1^ resulted in apparent rate constants of 0.06060, 0.06602, and 0.06574 min^−1^, respectively, over 2–30 min (Table S8). The similar rate constants obtained at 0.8 and 1.0 g L^−1^ indicate that the catalytic reaction approached a catalyst-loading plateau. Once sufficient catalytic sites were available, the amounts of PMS and SDZ relative to the catalyst became the main factors controlling the reaction rate. Catalyst-particle aggregation and nonproductive PMS consumption may further limit the benefit of increasing the catalyst loading.

The apparent rate constants at pH 2, 5, 7, and 9 were 0.05015, 0.06589, 0.07121, and 0.13388 min^−1^, respectively, over 2–20 min (Table S8). The highest apparent rate constant was obtained at pH 9, demonstrating that weakly alkaline conditions favored SDZ removal in the FeCo/NPC-800/PMS system. Solution pH influences PMS activation and the formation and interconversion of reactive species, thereby affecting SDZ degradation [56].

Increasing the PMS concentration from 0.1 to 0.5 mmol L^−1^ markedly accelerated SDZ removal. At PMS concentrations of 0.2 mmol L^−1^ or higher, the SDZ concentration rapidly decreased below the HPLC quantification limit, demonstrating rapid PMS activation and SDZ transformation. For the initial-SDZ-concentration series, apparent rate constants of 0.10543 and 0.06060 min^−1^ were obtained at 8 and 10 mg L^−1^, respectively. At initial SDZ concentrations of 4 and 6 mg L^−1^, SDZ decreased below the HPLC quantification limit within 10 and 20 min, respectively. These results demonstrate that lower initial SDZ concentrations favored more rapid removal because of the higher PMS-to-SDZ and accessible-site-to-SDZ ratios. At higher SDZ concentrations, SDZ and its transformation products compete for accessible surface sites. Adsorption of reaction intermediates and their accumulation within pores have been linked to activity loss in carbon-based PMS catalysts [57]. Such accumulation may cover Fe/Co-containing sites and obstruct pore channels, contributing to the slower SDZ removal alongside the lower PMS-to-SDZ ratio.

Five consecutive cycles were used to evaluate the reusability of FeCo/NPC-800 (Figure 8). The first cycle used 0.1 g of catalyst in 500 mL of a 10 mg L^−1^ SDZ solution. After each 60 min reaction, the catalyst was recovered by vacuum filtration, washed three times with 500 mL of distilled water per wash, and dried at 110 °C for 12 h. The mass of the recovered catalyst was used to adjust the volume of the freshly prepared SDZ/PMS solution in the subsequent cycle, thereby maintaining the catalyst concentration at 0.2 g L^−1^. After five cycles, FeCo/NPC-800 retained approximately 85% of its first-cycle SDZ-removal performance. The gradual decline in catalytic performance may reflect metal leaching, loss of fine catalyst particles during recovery, surface oxidation-state redistribution, and retention of adsorbed transformation products. Pore blockage has also been associated with declining removal performance during repeated use of waste-derived porous carbon catalysts for PMS activation [58]. This provides a basis for considering surface coverage and pore blockage as additional contributors to the decline observed here. After a separate standard 60 min reaction, the filtrate collected through a 0.22 μm membrane contained 1.632 ± 0.046 mg L^−1^ Fe, 5.127 ± 0.128 mg L^−1^ Co, and 0.0210 ± 0.0010 mg L^−1^ Zn, as determined by ICP-MS (Table S6). These concentrations correspond to approximately 3.95% and 10.07% of the initial Fe and Co inventories, respectively, at a catalyst loading of 0.2 g L^−1^. The release of Fe and Co decreases the number of metal-containing surface sites and may consequently contribute to the gradual loss of catalytic activity. Meanwhile, the released Fe and Co species may participate in solution-phase PMS activation during the reaction.

### 2.3. Analysis of ROS in the Catalytic Process

Reactive-species tests were conducted using 10 mg L^−1^ SDZ, 0.1 mmol L^−1^ PMS, 0.2 g L^−1^ FeCo/NPC-800, 0.05 mol L^−1^ MeOH or TBA, an initial pH of 7, and room temperature. TBA preferentially scavenges •OH, whereas MeOH quenches both •OH and SO_4_•^−^. MeOH produced substantially stronger inhibition of SDZ removal than TBA, demonstrating the participation of both SO_4_•^−^ and •OH and indicating that SO_4_•^−^ made the larger contribution to SDZ transformation under the tested conditions [59,60,61]. In a separate ESR experiment conducted in the FeCo/NPC-800/PMS system without SDZ, characteristic DMPO-adduct signals corresponding to •OH and SO_4_•^−^ were detected at 0, 2, 5, and 10 min. The signal evolution indicates rapid radical generation during the early reaction stage, followed by a gradual decrease associated with PMS consumption and spin-adduct decay. The DMPO-ESR results confirm the formation of both •OH and SO_4_•^−^ during PMS activation. TEMP-EPR measurements were also conducted in the FeCo/NPC-800/PMS system without SDZ. No characteristic 1:1:1 triplet signal corresponding to TEMP-derived TEMPO was detected at 0, 2, 5, or 10 min (Figure S8), indicating that ^1^O_2_ was not detected under the tested conditions. Taken together, the scavenging and ESR results demonstrate that SDZ transformation in the FeCo/NPC-800/PMS system proceeds predominantly through radical-mediated pathways involving SO_4_•^−^ and •OH, with SO_4_•^−^ serving as the principal detected reactive species (Figure 9).

### 2.4. Possible Degradation Pathways

TOC analysis shows that approximately 40% of organic carbon was removed after reaction, indicating partial mineralization of SDZ (Figure S6). Using HPLC–MS, the intermediates of SDZ degradation in the FeCo/NPC-800/PMS system were monitored at 10 and 40 min. Representative HPLC–MS spectra of the detected intermediates are provided in Figure S3. The possible degradation process is shown in Figure 10. Based on the detected mass-to-charge (*m*/*z*) signals and previously reported SDZ degradation pathways, three parallel transformation routes are proposed. In pathway I, oxidative conversion of the aniline amino group to a nitro group yields P1 (*m*/*z* 281). Subsequent cleavage of the labile sulfonamide bridge and sulfur dioxide extrusion yields P2 (*m*/*z* 217). In pathway II, attack at the sulfur–nitrogen bond leads to fragmentation into 4-nitrobenzenesulfonyl fragment P3 (*m*/*z* 186) and 2-aminopyrimidine P4 (*m*/*z* 96), while direct desulfonation produces P5 (*m*/*z* 187). In pathway III, hydroxylation of the pyrimidine ring yields monohydroxylated SDZ P6 (*m*/*z* 267). Further oxidation promotes ring opening and formation of the formyl-containing intermediate P7 (*m*/*z* 201), followed by smaller organic-acid fragments such as P8 (*m*/*z* 83). Continued oxidation may produce additional low-molecular-weight organic acids and partial mineralization.

## 3. Materials and Methods

### 3.1. Materials

The chemical reagents used in this study and their basic information are provided in the Supplementary Information (SI).

### 3.2. Preparation of Nitrogen-Stabilized Fe/Co-Containing Domains on N-Doped Porous Carbon

The synthesis method of FeCo/NPC-800 is shown in Figure 11. The detailed preparation process is provided in the Supplementary Information (SI).

### 3.3. Characterization

The characterization methods and instruments used in this study are detailed in the Supplementary Information (SI).

### 3.4. Batch Catalytic Degradation Experiments

Unless otherwise specified, batch experiments were conducted using 100 mL of 10 mg L^−1^ SDZ solution, 0.02 g of catalyst (0.2 g L^−1^), 0.1 mmol L^−1^ PMS, an initial pH of 7, and room temperature. PMS addition was defined as t = 0. Control experiments were conducted by omitting the catalyst or PMS as appropriate. Catalyst loadings of 0.2, 0.8, and 1.0 g L^−1^, initial pH values of 2, 5, 7, and 9, PMS concentrations of 0.1–0.5 mmol L^−1^, and initial SDZ concentrations of 4–10 mg L^−1^ were investigated by varying one factor while maintaining the remaining conditions constant. SDZ concentrations were determined by HPLC. Apparent pseudo-first-order kinetic constants were calculated by linear regression using ln(C/C_0_) = −kt + b over the specified early-time intervals, where k and b represent the apparent rate constant and intercept, respectively.

### 3.5. Reactive-Species, Intermediate, TOC, and Reusability Analyses

Scavenging tests used 0.05 mol L^−1^ TBA or MeOH under the standard SDZ/PMS/catalyst conditions. ESR measurements were performed separately in a system containing 0.2 g L^−1^ FeCo/NPC-800 and 0.1 mmol L^−1^ PMS at initial pH 7 and room temperature without SDZ. DMPO spectra were collected at 0, 2, 5, and 10 min. For the TEMP-EPR test, a 30 μL aliquot of this reaction mixture (concentrations specified before probe addition) was mixed with 50 μL of 100 mmol L^−1^ TEMP, and spectra were recorded in the dark at the same time points. For reusability tests, the initial cycle used 0.1 g catalyst in 500 mL of 10 mg L^−1^ SDZ solution. After each run, the catalyst was vacuum-filtered, washed three times with 500 mL distilled water per wash, dried at 110 °C for 12 h, weighed, and reused with a volume of fresh SDZ/PMS solution adjusted to maintain 0.2 g L^−1^ catalyst. HPLC–MS, TOC, ICP, and detailed EPR acquisition procedures are provided in the SI.

## 4. Conclusions

In this study, nitrogen-stabilized Fe/Co-containing domains supported on mushroom-residue-derived N-doped porous carbon were prepared through a dicyandiamide-assisted pyrolysis strategy. XRD identified locally ordered carbon and FeCo-related crystalline domains, while XAFS revealed the coexistence of metal–N/O and metal–metal coordination within small, structurally disordered Fe/Co-containing domains anchored to the N-doped carbon matrix. Direct comparison of NPC-800, Fe/NPC-800, Co/NPC-800, and FeCo/NPC-800 under identical PMS-activation conditions demonstrated the catalytic-performance advantage of simultaneous Fe and Co incorporation and supported the cooperative participation of Fe- and Co-containing sites in PMS activation. FeCo/NPC-800 exhibited an apparent pseudo-first-order rate constant of 0.06060 min^−1^ and achieved 91.2% aqueous SDZ removal within 60 min. Radical-scavenging and DMPO-ESR experiments confirmed the participation of SO_4_•^−^ and •OH, with SO_4_•^−^ serving as the principal detected reactive species. TEMP-EPR measurements showed no detectable ^1^O_2_ signal under the tested conditions, indicating that SDZ transformation was dominated by SO_4_•^−^ and •OH-mediated pathways. HPLC–MS analysis identified three principal transformation routes involving amino-group oxidation followed by sulfur dioxide extrusion, sulfur–nitrogen bond cleavage, and pyrimidine-ring opening. The approximately 40% TOC removal demonstrated partial mineralization of SDZ and its transformation products.

After five consecutive cycles, FeCo/NPC-800 retained approximately 85% of its first-cycle SDZ-removal performance. ICP-OES quantified Fe and Co contents of 20.638 and 25.449 wt%, respectively, in the catalyst, while ICP-MS detected 1.632 mg L^−1^ Fe and 5.127 mg L^−1^ Co in the filtrate after the standard 60 min reaction. The decrease in repeated-use performance was associated with metal release, surface oxidation-state redistribution, loss of catalytically active particles during recovery, and partial blockage of active sites and pore channels by adsorbed transformation products. The principal contribution of this study is the integration of mushroom-residue valorization, nitrogen-assisted stabilization of small Fe/Co-containing domains with XAFS-resolved metal–heteroatom and metal–metal coordination, and direct comparison with N-doped carbon and monometallic catalysts under identical reaction conditions. These findings establish an effective strategy for constructing waste-derived bimetallic carbon catalysts for PMS activation and antibiotic-contaminant transformation.

## Figures and Tables

**Figure 1:**
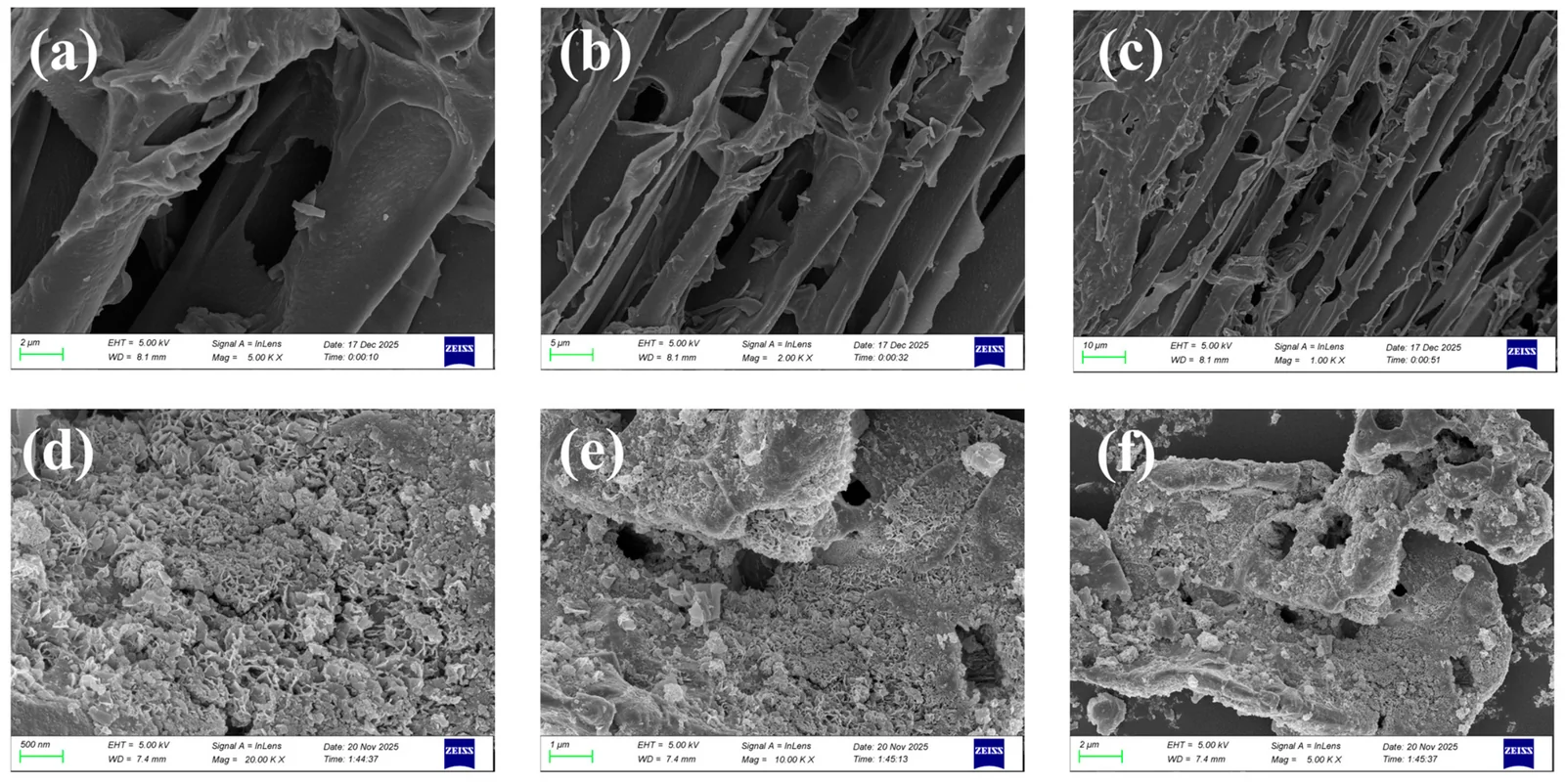
SEM images of MRB (**a**–**c**) and FeCo/NPC-800 (**d**–**f**).

**Figure 2:**
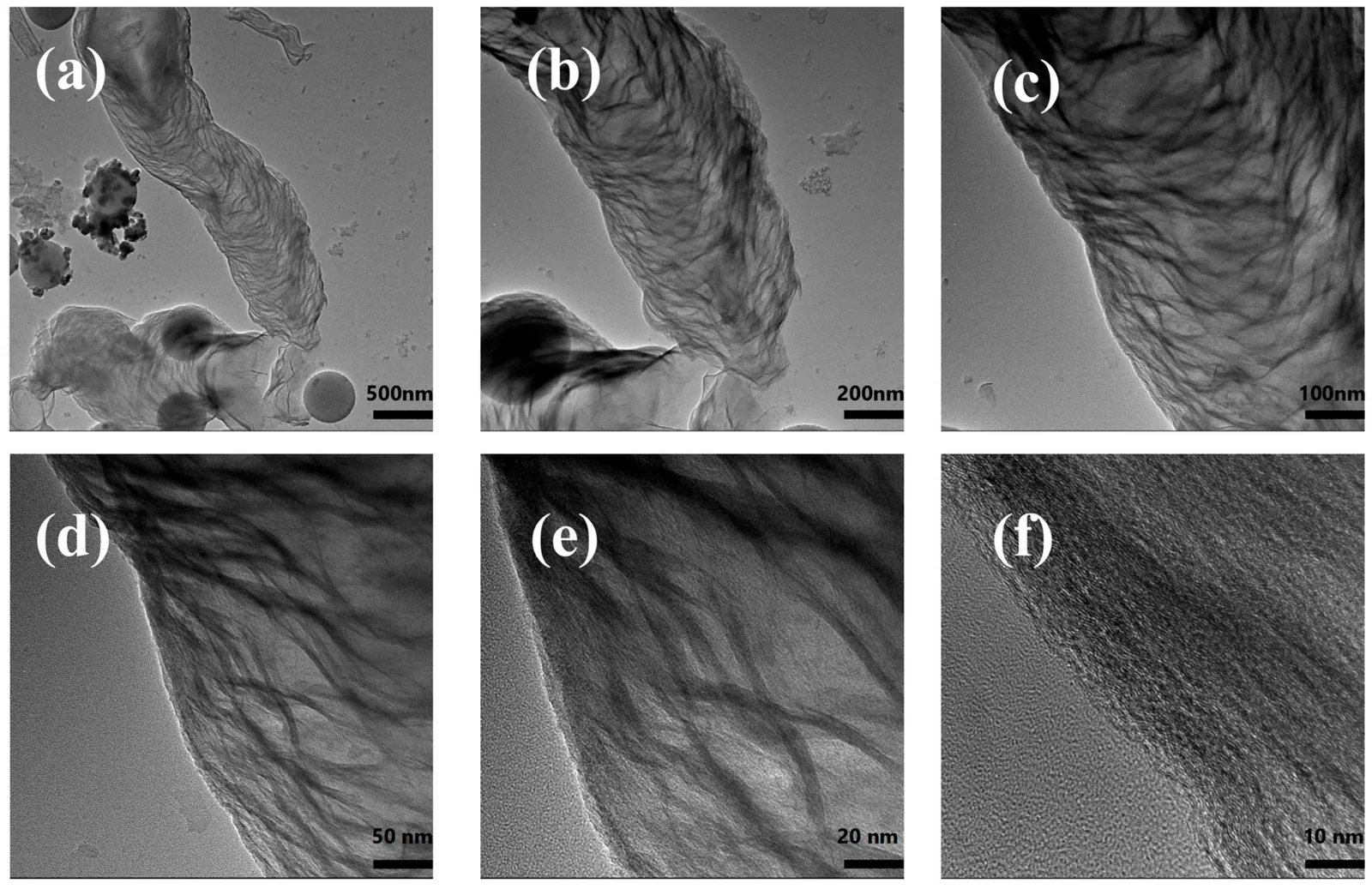
TEM images of the wrinkled and locally stacked carbon lamellae in FeCo/NPC-800 at different magnifications: (**a**) scale bar, 500 nm; (**b**) scale bar, 200 nm; (**c**) scale bar, 100 nm; (**d**) scale bar, 50 nm; (**e**) scale bar, 20 nm; (**f**) scale bar, 10 nm.

**Figure 3:**
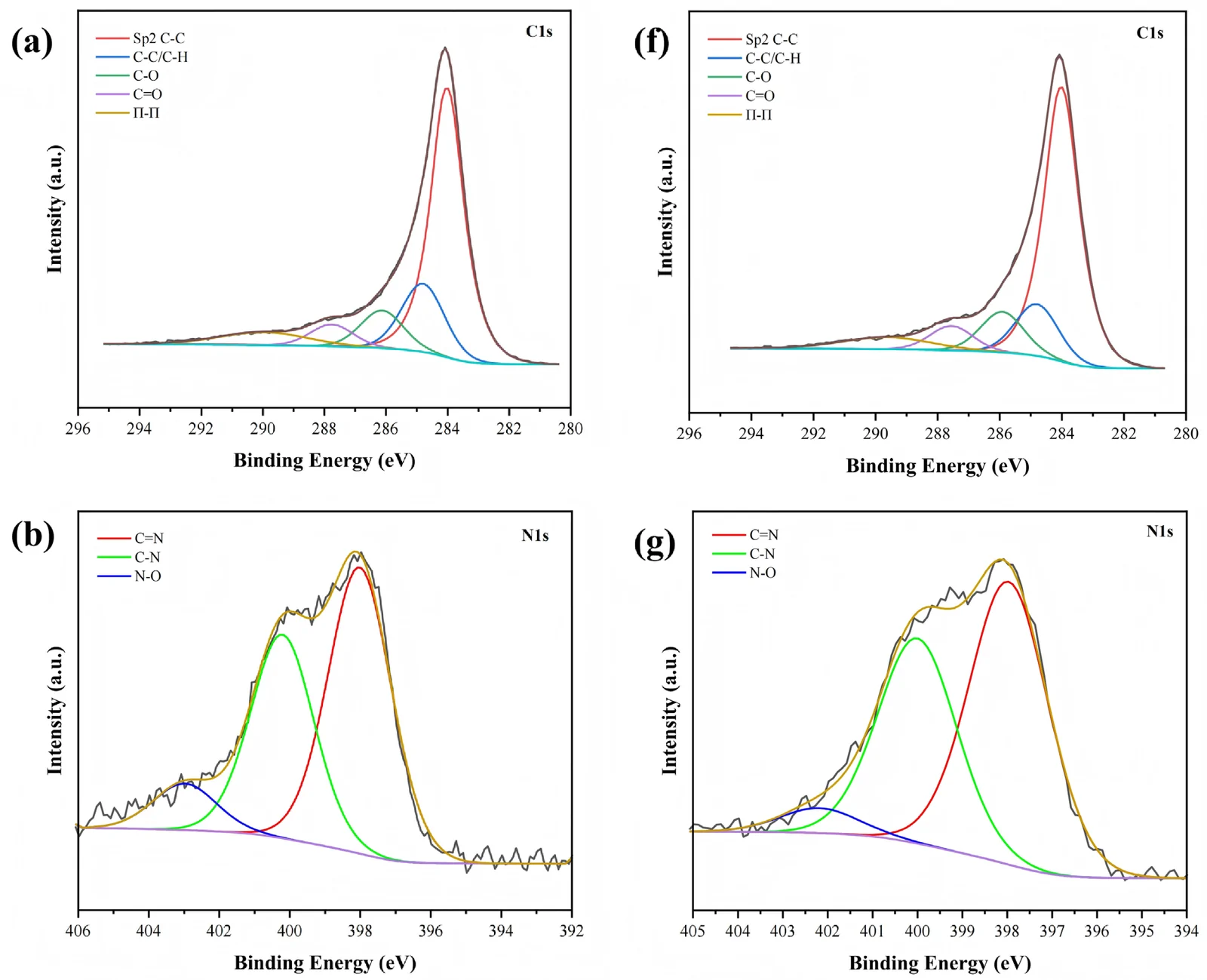

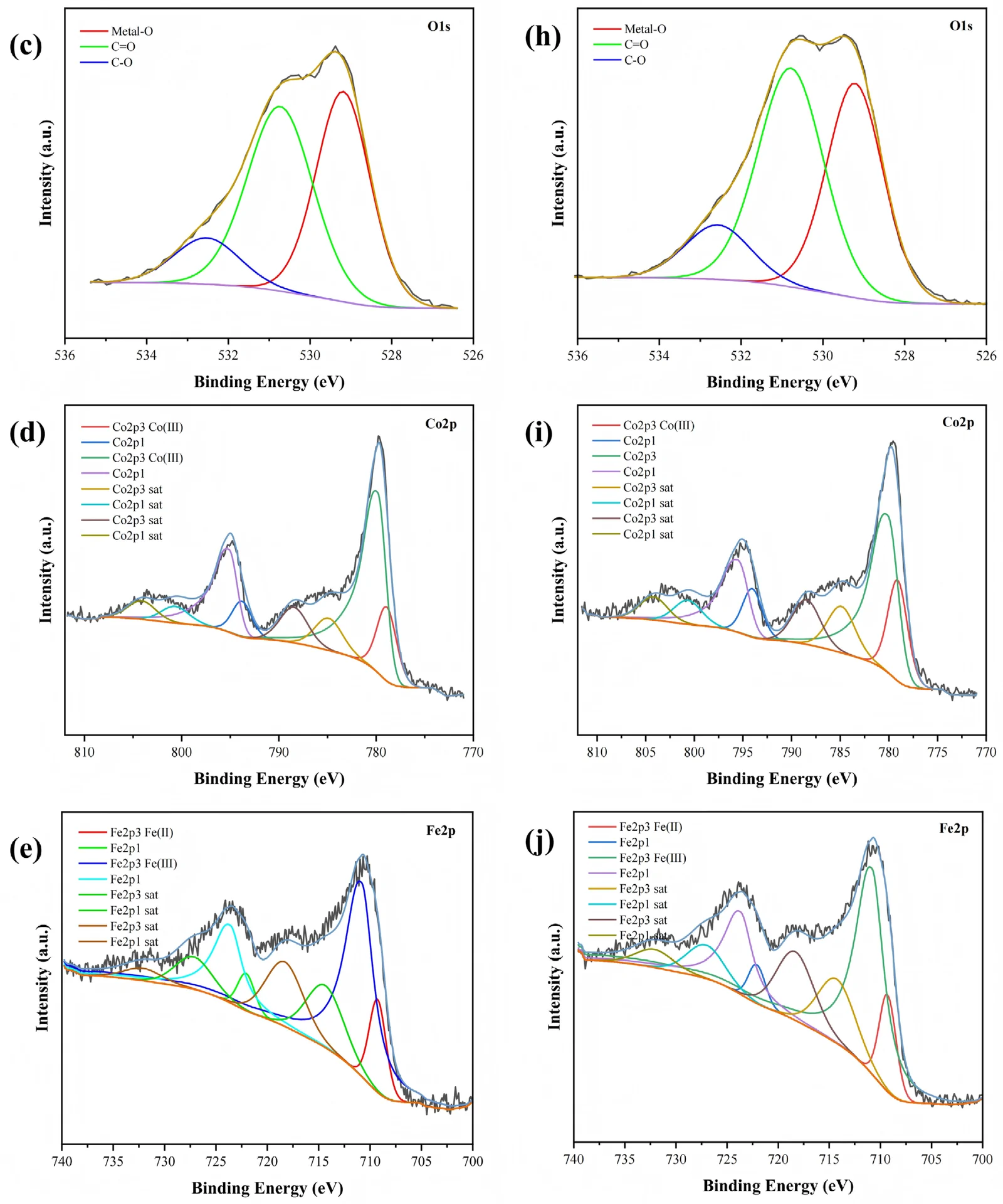
C 1s, N 1s, O 1s, Co 2p, and Fe 2p XPS spectra of FeCo/NPC-800 before reaction (**a**–**e**) and after reaction (**f**–**j**).

**Scheme 1:**
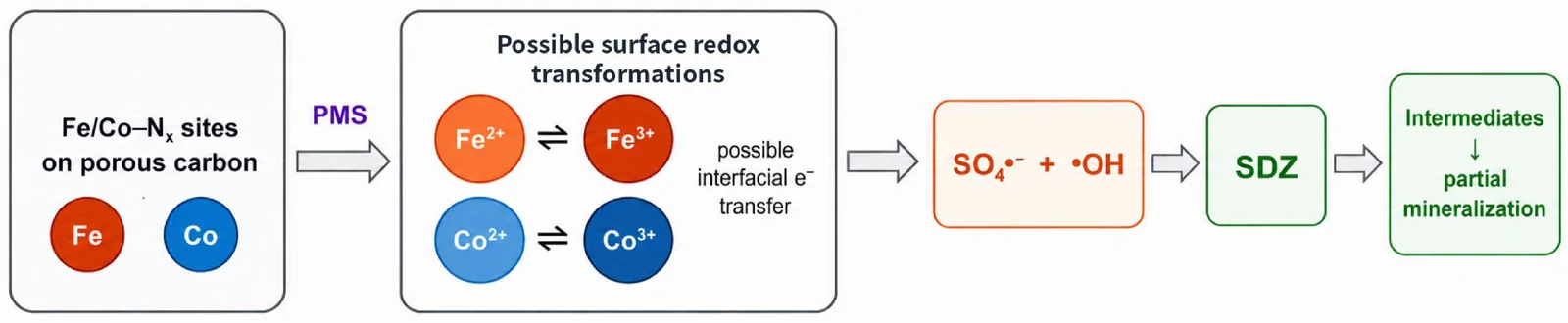
Proposed PMS activation pathways at Fe/Co-containing surface sites.

**Figure 4:**
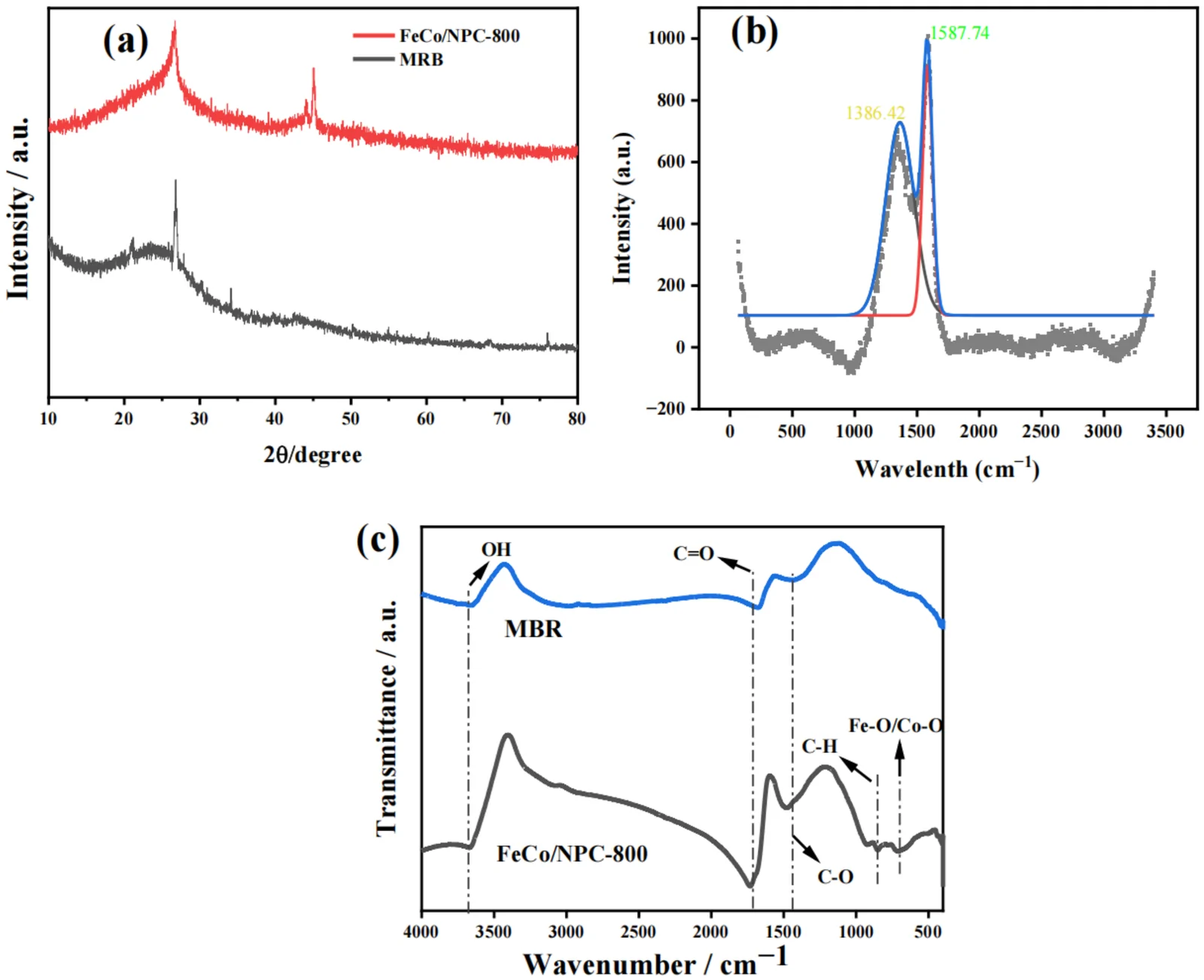
XRD patterns of MRB and FeCo/NPC-800 (**a**); Raman spectrum of FeCo/NPC-800 (**b**); FTIR spectra of FeCo/NPC-800 (**c**).

**Figure 5:**
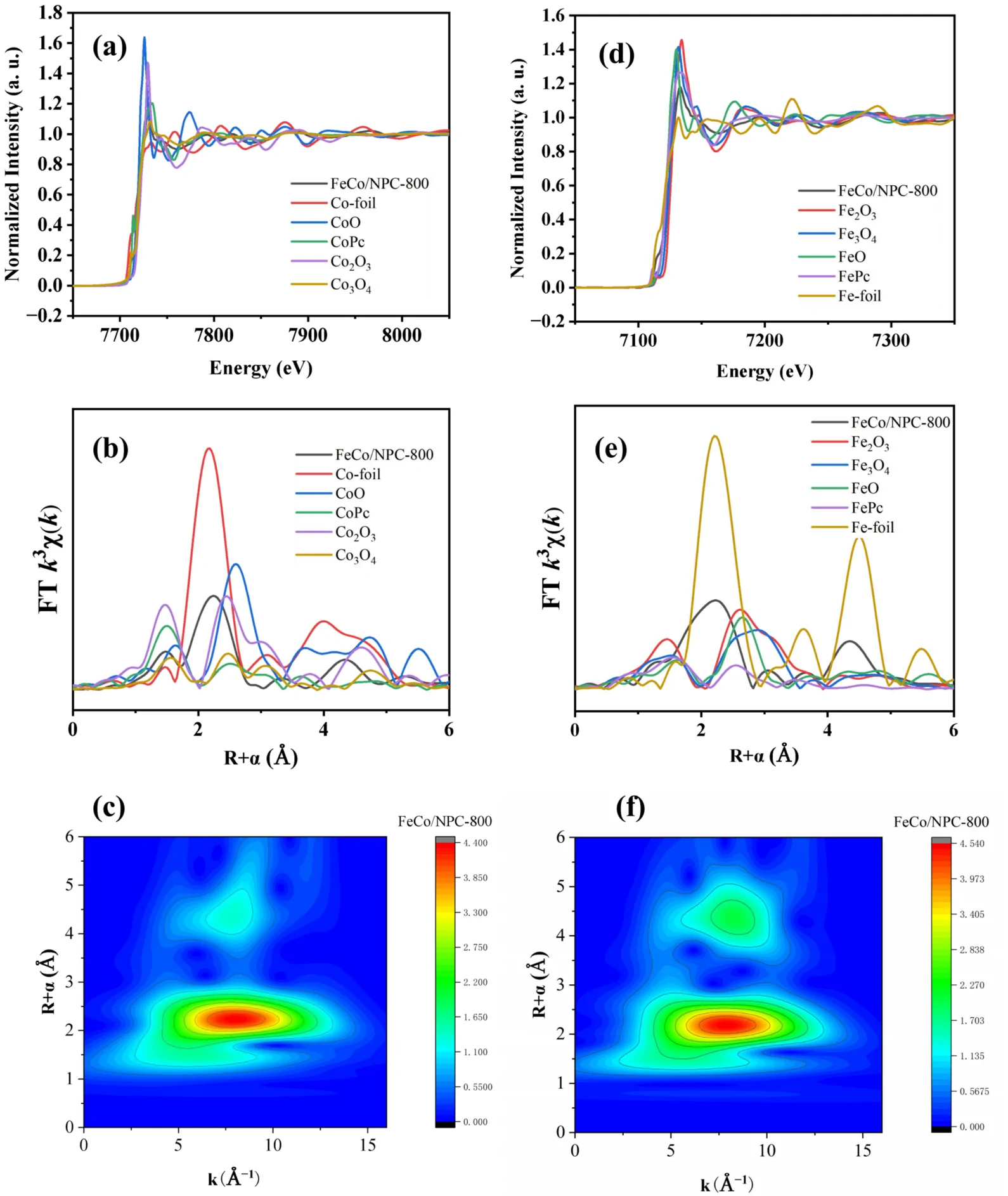
Co K-edge XANES spectrum (**a**), Co K-edge FT-EXAFS spectrum (**b**), and Co K-edge wavelet-transform map (**c**); Fe K-edge XANES spectrum (**d**), Fe K-edge FT-EXAFS spectrum (**e**), and Fe K-edge wavelet-transform map (**f**).

**Figure 6:**
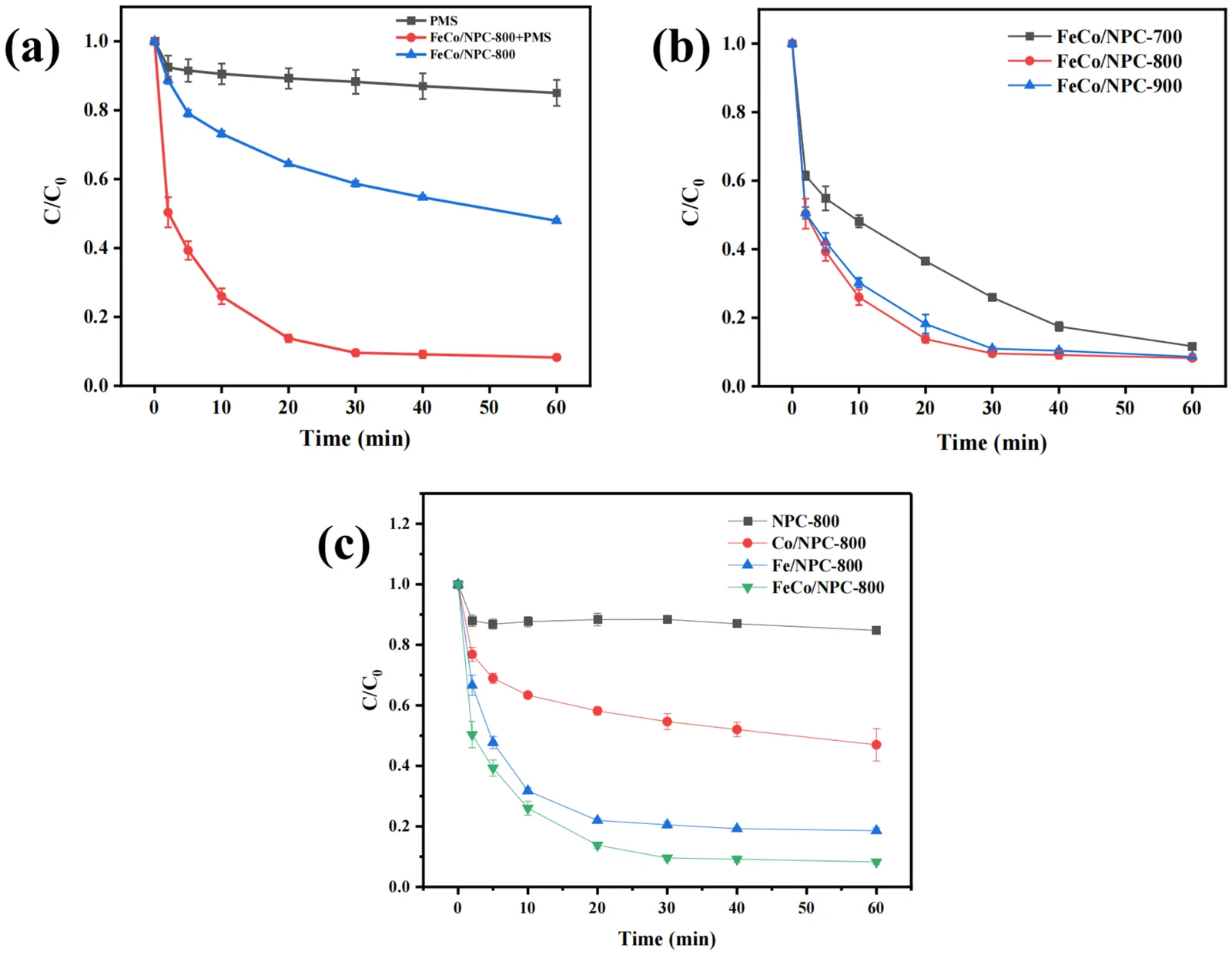
(**a**) SDZ removal by PMS alone, FeCo/NPC-800 alone, and FeCo/NPC-800/PMS; (**b**) effect of final pyrolysis temperature; (**c**) comparison of NPC-800, Fe/NPC-800, Co/NPC-800, and FeCo/NPC-800 in the presence of PMS. Condition: [SDZ]_0_ = 10 mg L^−1^, catalyst = 0.2 g L^−1^, [PMS]_0_ = 0.1 mmol L^−1^, initial pH = 7, room temperature.

**Figure 7:**
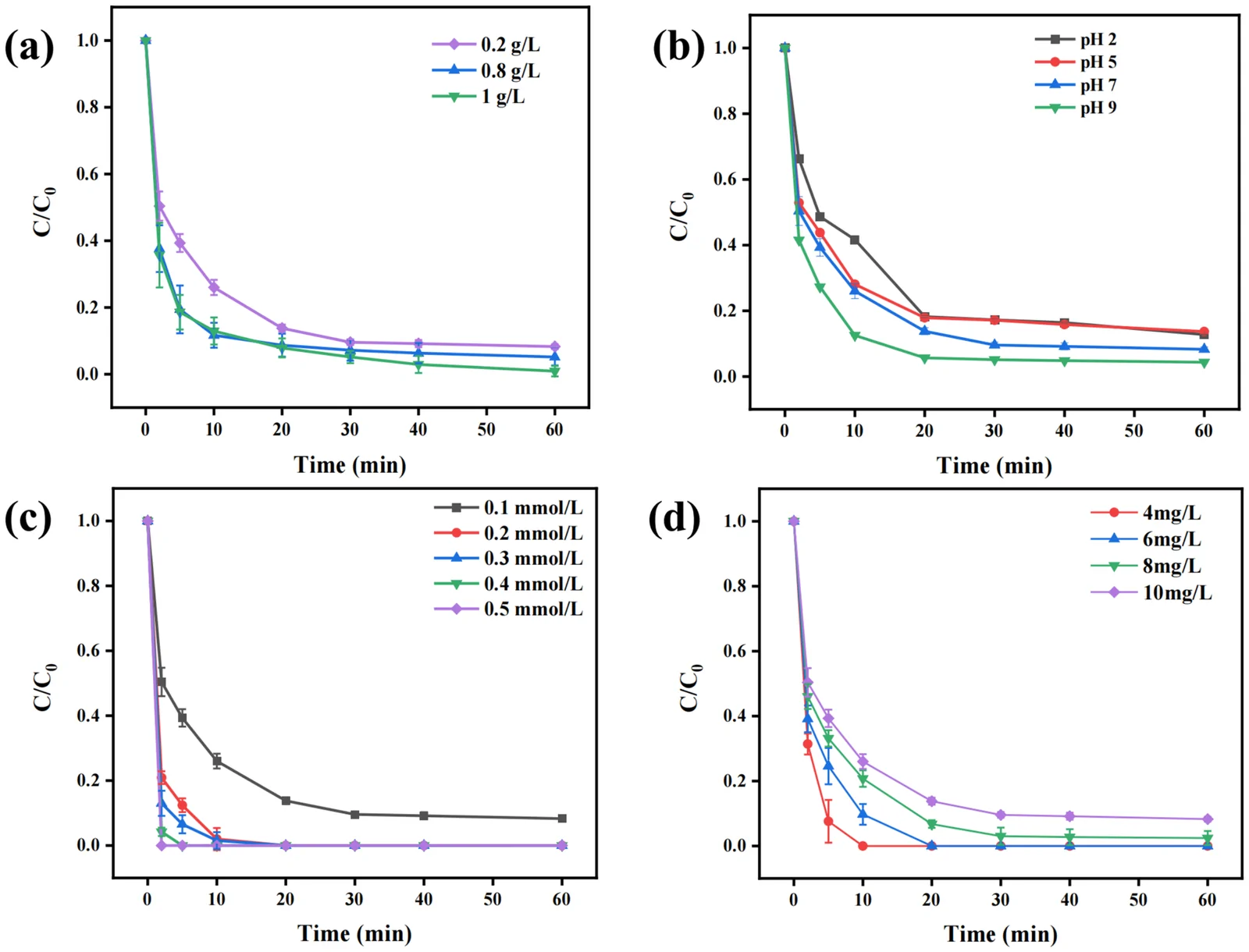
Effects of FeCo/NPC-800 dosage (**a**), initial pH (**b**), PMS concentration (**c**), and initial SDZ concentration (**d**) on SDZ removal.

**Figure 8:**
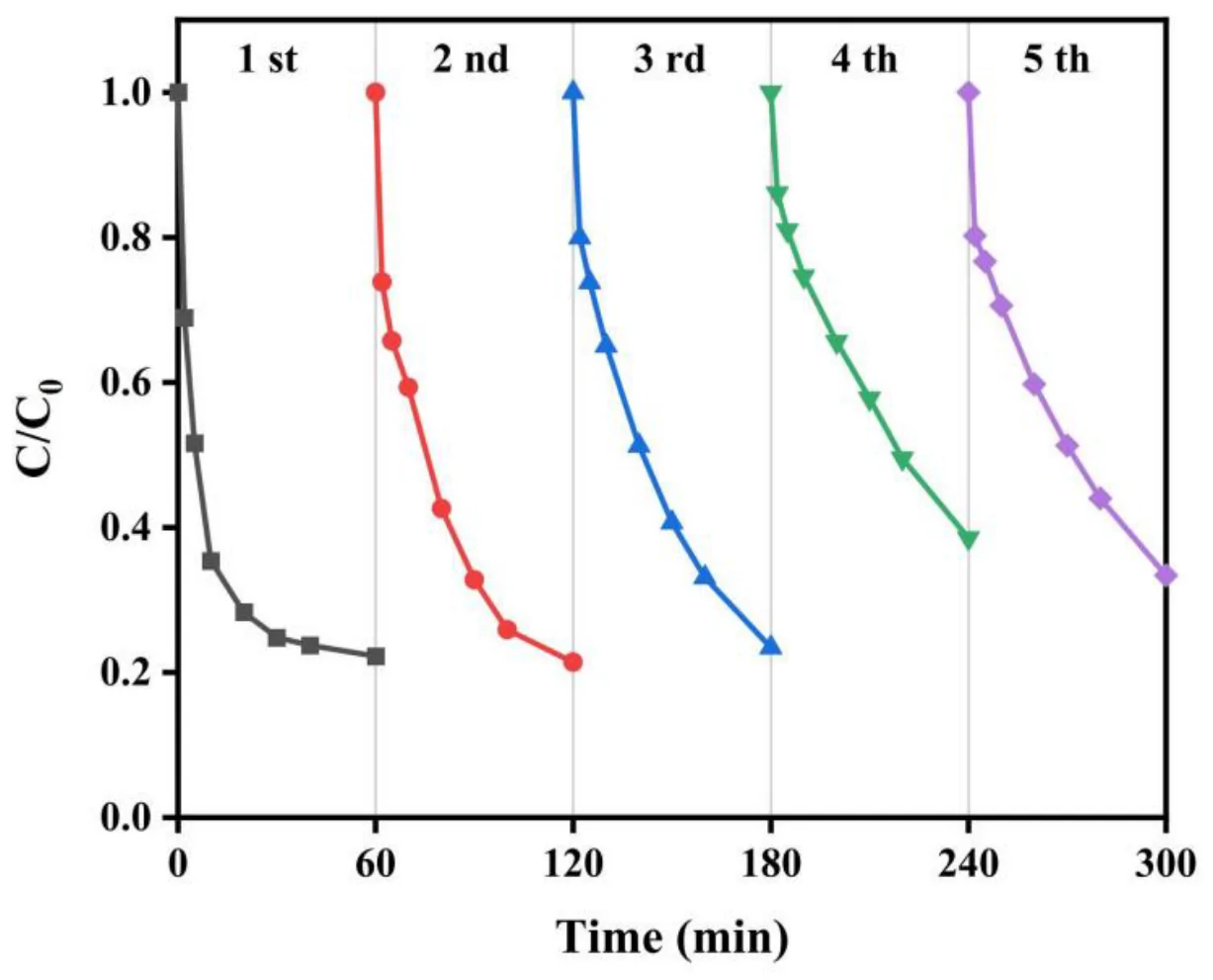
Reusability of FeCo/NPC-800 over five consecutive cycles.

**Figure 9:**
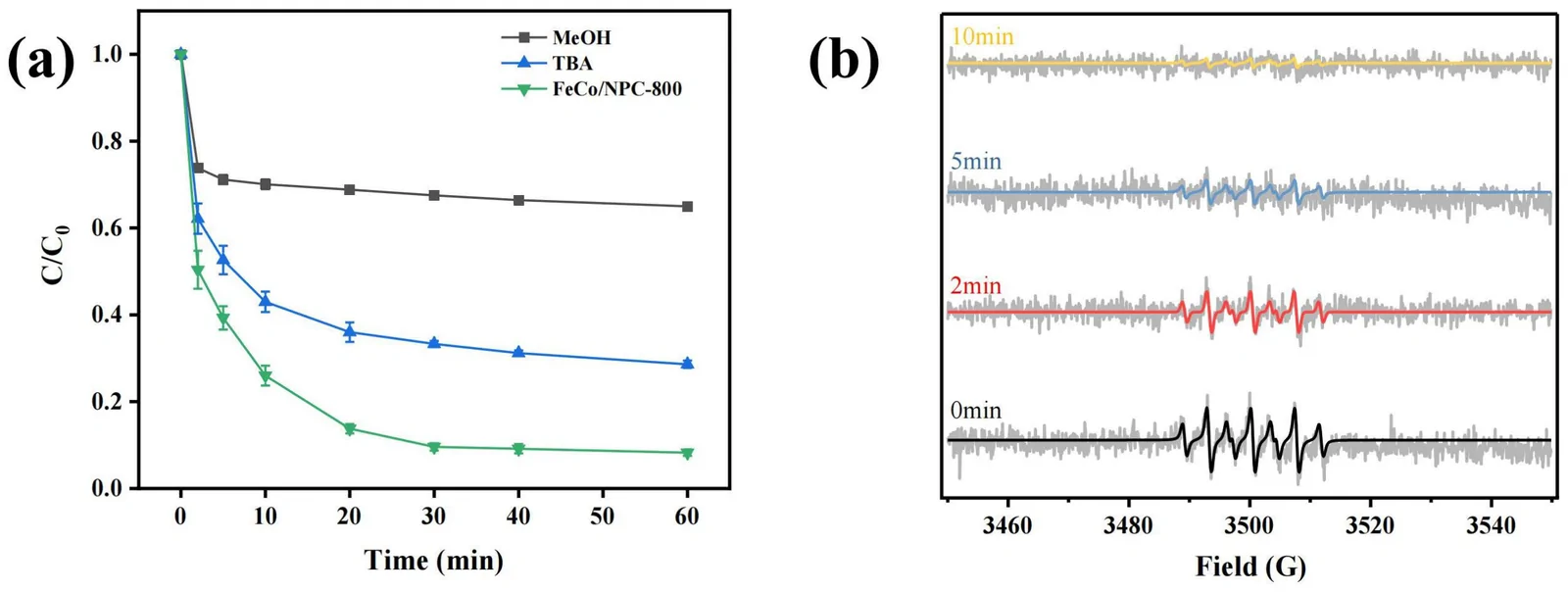
(**a**) Effects of 0.05 mol L^−1^ MeOH and TBA on SDZ removal ([SDZ]_0_ = 10 mg L^−1^, [PMS]_0_ = 0.1 mmol L^−1^, FeCo/NPC-800 = 0.2 g L^−1^, initial pH = 7, room temperature); (**b**) DMPO-ESR spectra collected at 0, 2, 5, and 10 min in a separate catalyst/PMS system without SDZ.

**Figure 10:**
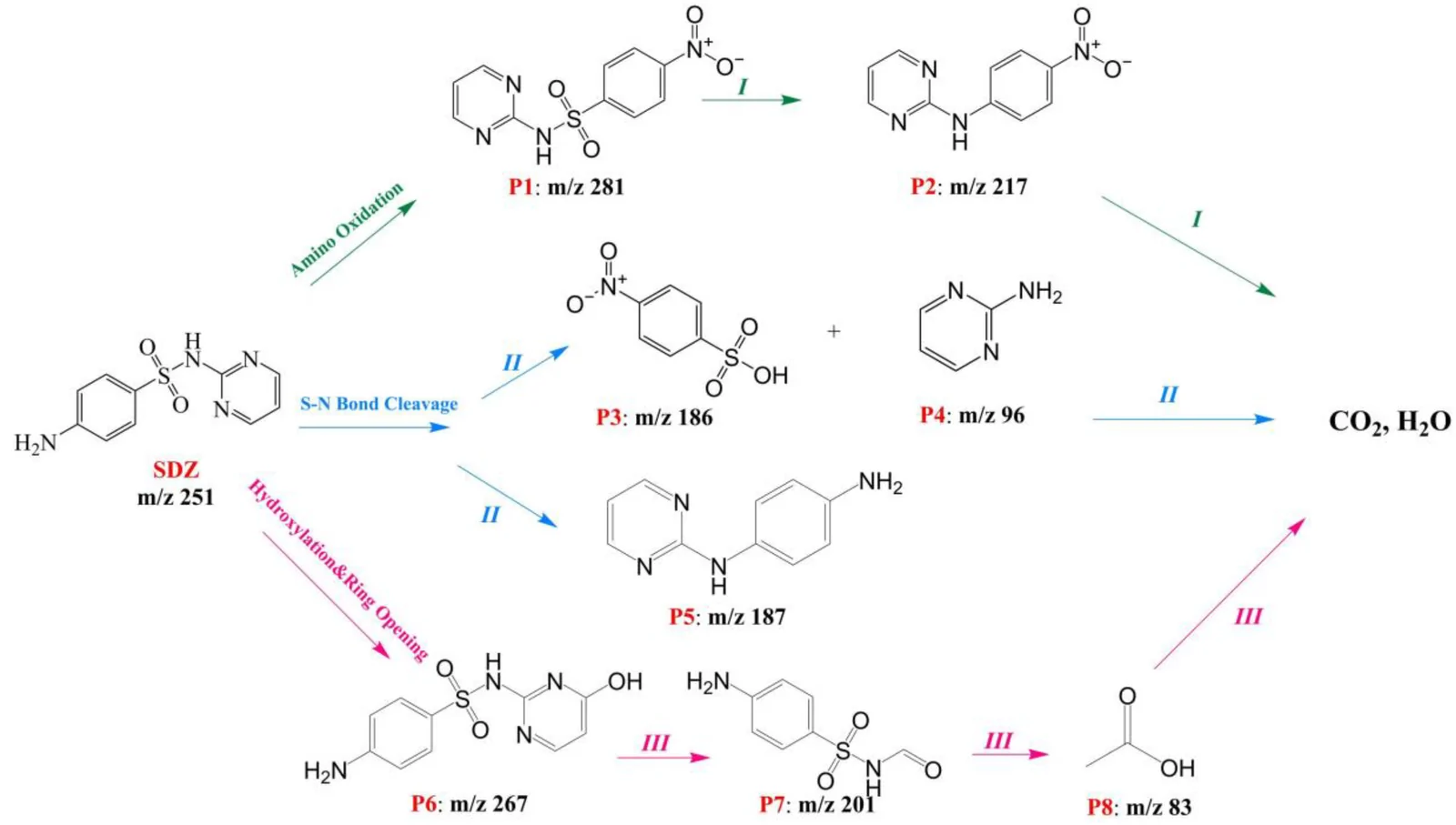
Possible degradation pathways of SDZ in the FeCo/NPC-800/PMS system.

**Figure 11:**
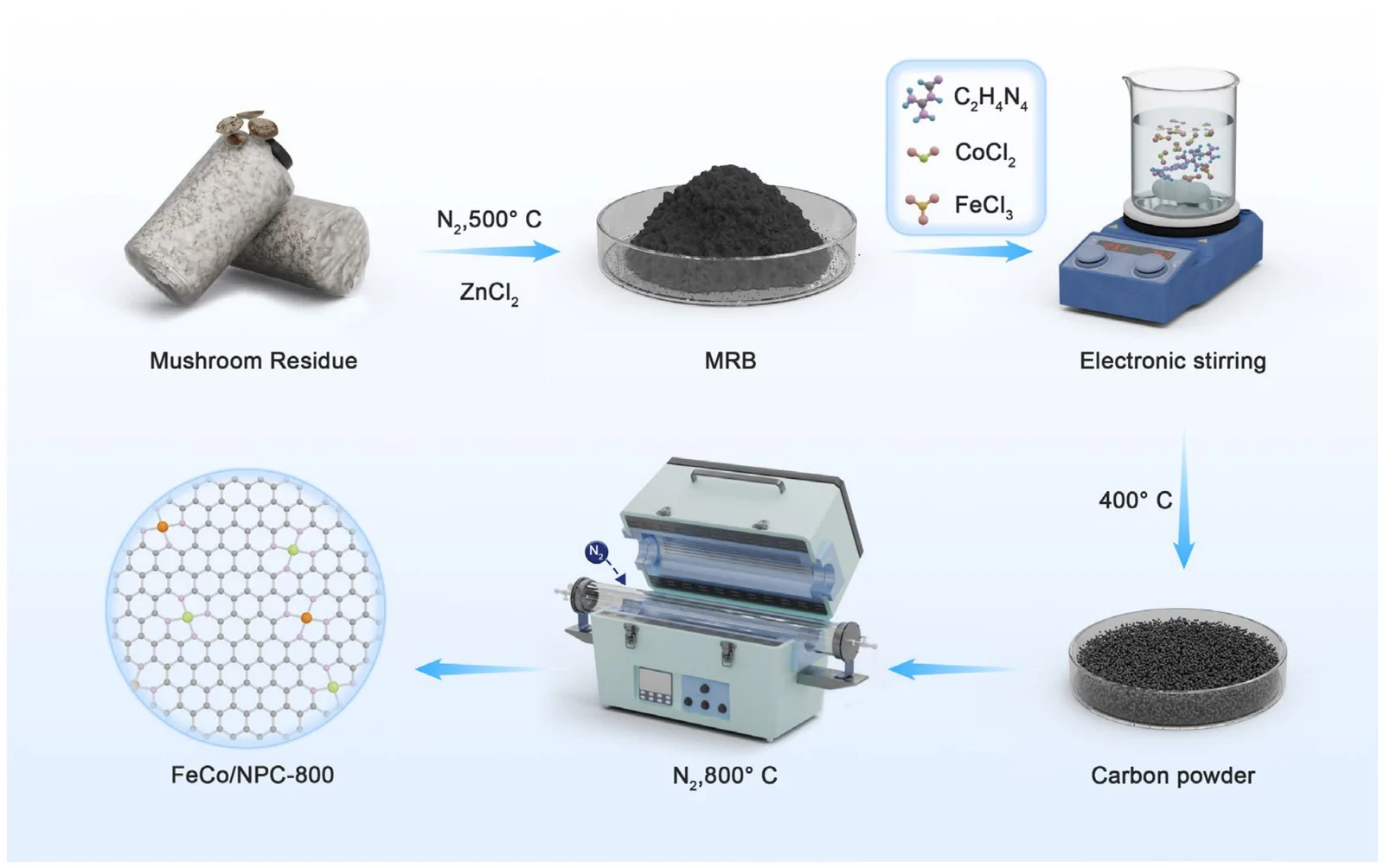
Schematic illustration of the preparation of nitrogen-stabilized ultrasmall Fe/Co-containing domains supported on mushroom-residue-derived N-doped porous carbon (FeCo/NPC-800).

## Data Availability

Data are contained within the article and Supplementary Materials.

## Supplementary Materials

The following supporting information can be downloaded at https://www.mdpi.com/article/10.3390/molecules31193392/s1: The Supporting Information, Tables S1–S9 and Figures S1–S8, includes details on materials, catalyst preparation, characterization, batch and kinetic procedures, reactive-species and reusability procedures, and ICP methods.
