# Supplementary material for: Nitrogen-Stabilized Fe–Co Nanoclusters Supported on Mushroom-Residue-Derived Porous Carbon for Peroxymonosulfate Activation Toward Sulfadiazine Degradation
Source: Molecules. 2026 Sep 24;31(19):3392. doi: 10.3390/molecules31193392 (PMC13666210; doi:10.3390/molecules31193392)
Supplement: Supplementary file 1 [file molecules-31-03392-s001.zip › molecules-4515444-supplementary.pdf]

Supplementary Information

Nitrogen-Stabilized Fe–Co Nanoclusters Supported on Mushroom-Residue-Derived Porous Carbon for Peroxymonosulfate Activation toward Sulfadiazine Degradation

Tianlong Zhang[1] , Yuxin Guo[1], Xiqi Xu[1], Changqing Hu[1], Bei Chu[1,*]

1 Ningbo Key Laboratory of Agricultural Germplasm Resources Mining and Environmental Regulation, College of Science and Technology, Ningbo University, Cixi 315300, China

* Corresponding author

E-mail addresses: chubei@nbu.edu.cn

1 Materials

Zinc chloride ($ZnCl_2$; 98% purity) was obtained from Shanghai Aladdin Biochemical Technology Co., Ltd., Shanghai, China. All other chemicals were supplied by Shanghai Macklin Biochemical Technology Co., Ltd., Shanghai, China, including potassium PMS ($2KHSO_5 \cdot KHSO_4 \cdot K_2SO_4$; 42%–45% purity), cobalt(II) chloride hexahydrate ($CoCl_2 \cdot 6H_2O$; 99% purity), ferric chloride hexahydrate ($FeCl_3 \cdot 6H_2O$; 99% purity), sulfadiazine (SDZ), dicyandiamide, hydrochloric acid (HCl), methanol (MeOH), tert-butanol (TBA), 5,5-dimethyl-1-pyrroline N-oxide (DMPO), and 2,2,6,6-tetramethylpiperidine (TEMP). All chemicals employed were analytical grade and used as received without purification.

2 Preparation of Fe–Co Nanocluster-Decorated N-Doped Porous Carbon

Pyrolysis and annealing were performed in a tube furnace (Tianjin Zhonghuan Electric Furnace Co., Ltd., Tianjin, China). Mushroom residue was dried at 110°C overnight and pyrolyzed at 400°C for 2 h (5°C min^{-1}) to obtain MR. $ZnCl_2$ was used as a chemical activating and pore-forming agent: MR and $ZnCl_2$ were physically mixed at a 1:1 mass ratio and heated at 500°C for 1 h under N_2 (5°C min^{-1}). The product was sequentially washed with 0.1 mol L^{-1} HCl, deionized water, and 40°C water until neutral, then dried at 110°C overnight to obtain MRB. ICP-OES later showed only 0.0152 ± 0.0001 wt% residual Zn in FeCo/NPC-800 (Table S6).

For FeCo/NPC-800, 5 g dicyandiamide was dissolved in 100 mL of a mixed aqueous solution containing 0.1 mol L^{-1} $CoCl_2 \cdot 6H_2O$ and 0.1 mol L^{-1} $FeCl_3 \cdot 6H_2O$ (10 mmol each, corresponding to approximately 2.379 g $CoCl_2 \cdot 6H_2O$ and 2.703 g $FeCl_3 \cdot 6H_2O$; nominal Fe:Co molar ratio 1:1). MRB (0.5 g) was added, stirred for 1 h, and dried at 90°C for 12 h. The precursor was ground, pre-carbonized at 400 °C for 2 h (5°C min^{-1}), annealed at 800°C for 1 h under N_2 at the same heating rate, alternately washed with 1 mol L^{-1} HCl and deionized water until neutral, and dried at 110°C overnight. FeCo/NPC-700 and FeCo/NPC-900 were prepared analogously using final annealing temperatures of 700 and 900°C. NPC-800, Fe/NPC-800, and Co/NPC-800 controls were prepared under the same thermal protocol by omitting both metal salts or using only the Fe or Co precursor, respectively.

3 Characterization

Morphology and carbon architecture were examined by SEM (Sigma 300; Carl Zeiss Microscopy GmbH, Jena, Germany) and TEM (JEM-2100F; JEOL Ltd., Akishima, Japan). Textural properties were determined from N_2 adsorption–desorption isotherms at –196°C using an ASAP 2020 analyzer (Micromeritics Instrument Corporation, Norcross, GA, USA). XAFS data were collected at the BL14W1 beamline of the Shanghai Synchrotron Radiation Facility, and XRD patterns were obtained using an Ultima IV diffractometer (Rigaku Corporation, Akishima, Japan). XPS spectra were acquired using a K-Alpha XPS instrument (Thermo Fisher Scientific Inc., Waltham, MA, USA) and processed in Avantage software (version 6.6085) by nonlinear least-squares fitting with mixed Gaussian – Lorentzian components. A Shirley background was applied, and binding energies were referenced to the C 1s C–C/C–H component at 284.8 eV. Corresponding $2p_{3/2}$ and $2p_{1/2}$ components were assigned as chemically consistent chemical-state pairs; the final fitted FWHM values of each pair were equal or closely comparable (Table S5). Shake-up satellites in the Fe 2p and Co 2p regions were included in the overall envelopes but excluded from oxidation-state proportion calculations. SDZ was quantified by HPLC (1260 Infinity II; Agilent Technologies, Inc., Santa Clara, CA, USA), and transformation products were characterized by HPLC-MS (1290 UPLC-QTOF 6550; Agilent Technologies, Inc., Santa Clara, CA, USA). Total organic carbon (TOC) was determined using a TOC-L CPH analyzer (Shimadzu Corporation, Kyoto, Japan).

4 Batch Catalytic Experiments and Kinetic Analysis

Unless otherwise specified, 0.02 g catalyst was dispersed in 100 mL of 10 mg L^{-1} SDZ solution (0.2 g L^{-1} catalyst), the initial pH was 7, and oxidation was initiated by adding PMS to 0.1 mmol L^{-1} at room temperature. PMS addition defined t = 0. Catalyst-free, PMS-free, and material-control tests were conducted under otherwise identical conditions. Catalyst loading (0.2, 0.8, and 1.0 g L^{-1}), initial pH (2, 5, 7, and 9), PMS concentration (0.1–0.5 mmol L^{-1}), and initial SDZ concentration (4, 6, 8, and 10 mg L^{-1}) were varied separately. Apparent pseudo-first-order constants were obtained from $\ln(C/C_0) = -kt + b$ over the intervals listed in Tables S7 and S8, where k is the apparent rate constant. HPLC records with no detectable SDZ peak

were recorded as below the quantification limit and were excluded from logarithmic fitting; no rate constant was reported when fewer than three quantifiable nonzero points remained.

5 Reactive-Species and Reusability Experiments

Scavenging experiments used 10 mg L^{-1} SDZ, 0.1 mmol L^{-1} PMS, 0.2 g L^{-1} FeCo/NPC-800, and 0.05 mol L^{-1} MeOH or TBA at initial pH 7 and room temperature. ESR measurements were conducted separately without SDZ in a system containing 0.2 g L^{-1} FeCo/NPC-800 and 0.1 mmol L^{-1} PMS at initial pH 7 and room temperature. DMPO spectra were collected at 0, 2, 5, and 10 min. To examine $^1O_2$, a 30 μL aliquot of this reaction mixture (concentrations specified before probe addition) was mixed with 50 μL of 100 mmol L^{-1} TEMP, transferred to a capillary tube, sealed, and measured in the dark at 0, 2, 5, and 10 min using an EMXplus-6/1 spectrometer (Bruker BioSpin GmbH, Rheinstetten, Germany). The principal EPR acquisition parameters were a center field of 3500 G, sweep width of 100 G, microwave frequency of approximately 9.8323 GHz, microwave power of 6.325 mW, modulation amplitude of 1.0 G, modulation frequency of 100 kHz, sweep time of 30 s, receiver gain of 30 dB, and 1000 data points. Four scans were accumulated for the 0, 2, and 5 min spectra, and five scans for the 10 min spectrum. The nominal TEMP concentration after mixing was 62.5 mmol L^{-1} (assuming additive volumes). The plotted spectra are the exported data without further smoothing or baseline adjustment.

For cycling tests, the first run used 0.1 g FeCo/NPC-800 in 500 mL of 10 mg L^{-1} SDZ solution. After each 60 min run, the catalyst was recovered by vacuum filtration through ordinary filter paper, washed three times with 500 mL distilled water per wash, and dried at 110°C for 12 h. The recovered catalyst was weighed, and the volume of freshly prepared SDZ/PMS solution in the next run was adjusted to maintain 0.2 g L^{-1} catalyst while keeping the SDZ and PMS concentrations unchanged.

6 ICP-OES and ICP-MS Measurements

Solid FeCo/NPC-800 was subjected to microwave digestion and analyzed for Fe, Co, and Zn using a 5110 ICP-OES instrument (Agilent Technologies, Inc., Santa Clara, CA, USA). For dissolved-metal analysis, a separate standard 60 min reaction used 100 mL of 10 mg L^{-1} SDZ, 0.02 g FeCo/NPC-800, 0.1 mmol L^{-1} PMS, initial pH 7, and room temperature. The suspension

was passed through a 0.22 μm membrane; the filtrate was acid-treated by the analytical service provider and analyzed using a 7800 ICP-MS instrument (Agilent Technologies, Inc., Santa Clara, CA, USA). Results are reported as mean ± SD of triplicate instrumental readings from the same prepared sample or filtrate.

Table S1. Textural properties of the FeCo/NPC-T samples.

	unit	FeCo/NPC-700	FeCo/NPC-800	FeCo/NPC-900
**S_{BET}**	m^2/g	41.59	423.5	1289
**V_{total}**	cm^3/g	0.0313	0.3403	0.9148
**V_{micro}**	cm^3/g	0.0150	0.1522	0.4895
**BJH adsorption cumulative pore volume**	cm^3/g	0.008636	0.1701	0.3862
**Average adsorption pore diameter**	Å	30.10	32.14	28.91
**Maximum pore volume at P/Po**	cm^3/g	0.01896	0.1978	0.5830

Table S2 . EXAFS fitting parameters at the Co K-edge (S_0^2 = 0.90 from Co foil). CoPc denotes cobalt phthalocyanine; FeCo/NPC-800 denotes the catalyst measured at the Co K-edge.

<table>
<tr><th></th><th>shell</th><th>CN^a</th><th>R^b(Å)</th><th>σ^{2c}(Å²)</th><th>ΔE₀^d(eV)</th><th>R factor</th></tr>
<tr><td>Co-foil</td><td>Co-Co</td><td>12.0</td><td>2.49±0.01</td><td>0.0063</td><td>6.9±0.3</td><td>0.0007</td></tr>
<tr><td rowspan="2">CoPc</td><td>Co-N</td><td>4.2±0.4</td><td>1.90±0.01</td><td>0.0010</td><td rowspan="2">7.3±1.3</td><td rowspan="2">0.0191</td></tr>
<tr><td>Co-N-C</td><td>2.1±0.1</td><td>2.92±0.01</td><td>0.0030</td></tr>
<tr><td rowspan="3">FeCo/NPC-800</td><td>Co-N1</td><td>2.0±0.1</td><td>1.96±0.01</td><td>0.0030</td><td rowspan="3">8.1±3.3</td><td rowspan="3">0.0169</td></tr>
<tr><td>Co-N2</td><td>2.3±0.3</td><td>2.13±0.01</td><td>0.0042</td></tr>
<tr><td>Co-Fe</td><td>4.3±0.3</td><td>2.52±0.01</td><td>0.0051</td></tr>
<tr><td rowspan="2">CoO</td><td>Co-O</td><td>2.9±0.7</td><td>2.09±0.01</td><td>0.0177</td><td rowspan="2">–2.3±0.8</td><td rowspan="2">0.0094</td></tr>
<tr><td>Co-Co</td><td>12.3±0.3</td><td>3.01±0.01</td><td>0.0095</td></tr>
<tr><td rowspan="3">Co₂O₃</td><td>Co-O</td><td>4.6±0.5</td><td>1.92±0.08</td><td>0.0025</td><td rowspan="3">–4.8±1.0</td><td rowspan="3">0.0097</td></tr>
<tr><td>Co-Co1</td><td>1.4±0.2</td><td>2.88±0.01</td><td>0.0075</td></tr>
<tr><td>Co-Co2</td><td>2.0±0.1</td><td>3.38±0.01</td><td>0.0032</td></tr>
<tr><td rowspan="3">Co₃O₄</td><td>Co-O</td><td>4.2±0.8</td><td>1.93±0.07</td><td>0.0020</td><td rowspan="3">–3.2±2.5</td><td rowspan="3">0.0131</td></tr>
<tr><td>Co-Co1</td><td>3.4±0.7</td><td>2.85±0.03</td><td>0.0012</td></tr>
<tr><td>Co-Co2</td><td>10.6±1.5</td><td>3.17±0.04</td><td>0.0085</td></tr>
</table>

[a]*CN*: coordination numbers; [b]*R*: bond distance; [c]σ^2: Debye-Waller factors; [d] ΔE_0: the inner potential correction. R factor: goodness of fit. Error bounds that characterize the structural parameters obtained by EXAFS spectroscopy were estimated as CN±20%; R ± 1%; σ^2 ± 20%.

Table S3 . EXAFS fitting parameters at the Fe K-edge (S_0^2 = 0.85 from Fe foil). FePc denotes iron phthalocyanine; FeCo/NPC-800 denotes the catalyst measured at the Fe K-edge.

	shell	CN[a]	R^b(Å)	σ^{2c}(Å^2)	ΔE_0^d(eV)	R factor
**Fe-foil**	Fe-Fe1	8.0±0.1	2.83±0.02	0.0017	-7.8±2.6	0.0094
	Fe-Fe2	6.0±0.1	2.43±0.01	0.0020		
**FeO**	Fe-O	6.0±0.1	2.11±0.01	0.0161	–0.1±0.8	0.0056
	Fe-Fe	12.0±0.1	3.07±0.01	0.0132		
**Fe_2O_3**	Fe-O1	1.0±0.1	1.92±0.03	0.0013	3.4±2.5	0.0098
	Fe-O2	5.2±0.2	2.10±0.02	0.0181		
	Fe-Fe1	3.0±0.1	3.00±0.04	0.0017		
	Fe-Fe2	3.0±0.1	3.33±0.01	0.0058		
**Fe_3O_4**	Fe-O1	1.0±0.1	1.84±0.01	0.0028	–4.7±3.2	0.0103
	Fe-O2	1.5±0.2	2.00±0.01	0.0075		
	Fe-Fe1	3.2±0.1	3.02±0.02	0.0042		
	Fe-Fe2	4.0±0.1	3.47±0.03	0.0171		
**FePc**	Fe-N	4.0±0.1	1.98±0.01	0.0075	3.1±2.5	0.0068
	Fe-C	2.0±0.1	2.99±0.01	0.0101		
FeCo/NPC-800	Fe-N1	2.0±0.1	1.93±0.01	0.0014	9.0±3.1	0.0184
	Fe-N2	2.3±0.3	2.10±0.01	0.0030		
	Fe-Co	4.0±0.1	2.52±0.01	0.0030		

[a]*CN*: coordination numbers; [b]*R*: bond distance; [c]σ^2: Debye-Waller factors; [d] ΔE_0: the inner potential correction. R factor: goodness of fit. Error bounds that characterize the structural parameters obtained by EXAFS spectroscopy were estimated as CN±20%; R ± 1%; σ^2 ± 20%.

Table S4. XPS fitting parameters for the C 1s, N 1s, and O 1s regions of fresh and used FeCo/NPC-800.

Sample	Region	Assignment	Binding energy (eV)	FWHM (eV)
Fresh	C 1s	Sp^2 C–C	284.00	1.28
Fresh	C 1s	C–C/C–H	284.80	1.64
Fresh	C 1s	C–O/C–N	286.13	1.64
Fresh	C 1s	C=O	287.76	1.64
Fresh	C 1s	π–π^*/O–C=O	289.97	3.37
Fresh	N 1s	C=N	398.02	2.12
Fresh	N 1s	C–N	400.21	2.12
Fresh	N 1s	N–O	402.98	2.12
Fresh	O 1s	Metal–O	529.19	1.56
Fresh	O 1s	C=O	530.73	1.90
Fresh	O 1s	C–O	532.53	1.90
Used	C 1s	Sp^2 C–C	284.00	1.34
Used	C 1s	C–C/C–H	284.80	1.64
Used	C 1s	C–O/C–N	285.91	1.64
Used	C 1s	C=O	287.55	1.64
Used	C 1s	π–π^*/O–C=O	289.62	3.37
Used	N 1s	C=N	397.97	2.08
Used	N 1s	C–N	400.00	2.08

Sample	Region	Assignment	Binding energy (eV)	FWHM (eV)
Used	N 1s	N–O	402.19	2.08
Used	O 1s	Metal–O	529.21	1.63
Used	O 1s	C=O	530.78	1.89
Used	O 1s	C–O	532.57	1.89

Table S5. XPS fitting parameters and satellite treatment for the Fe 2p and Co 2p regions.

Sample	Region	Component	Binding energy (eV)	FWHM (eV)	Treatment
Fresh	Fe 2p	Fe(II) $2p_{3/2}$	709.26	1.94	Oxidation-state component
Fresh	Fe 2p	Fe(II) $2p_{1/2}$	722.06	1.94	Oxidation-state component
Fresh	Fe 2p	Fe(III) $2p_{3/2}$	710.81	3.34	Oxidation-state component
Fresh	Fe 2p	Fe(III) $2p_{1/2}$	723.61	3.32	Oxidation-state component
Fresh	Fe 2p	Satellite 1 ($2p_{3/2}$ / $2p_{1/2}$)	714.37 / 727.17	4.33 / 4.33	Included in envelope; excluded from state proportions
Fresh	Fe 2p	Satellite 2 ($2p_{3/2}$ / $2p_{1/2}$)	718.30 / 732.10	4.33 / 4.33	Included in envelope; excluded from state proportions
Fresh	Co 2p	Co(III) $2p_{3/2}$ / $2p_{1/2}$	778.90 / 793.80	2.38 / 2.38	Oxidation-state components
Fresh	Co 2p	Co(II) $2p_{3/2}$ / $2p_{1/2}$	779.85 / 795.15	2.94 / 2.94	Oxidation-state components
Fresh	Co 2p	Satellite 1 ($2p_{3/2}$ / $2p_{1/2}$)	784.88 / 800.67	3.37 / 3.37	Included in envelope; excluded from state proportions

Sample	Region	Component	Binding energy (eV)	FWHM (eV)	Treatment
Fresh	Co 2p	Satellite 2 ($2p_{3/2}$ / $2p_{1/2}$)	788.47 / 804.12	3.37 / 3.37	Included in envelope; excluded from state proportions
Used	Fe 2p	Fe(II) $2p_{3/2}$	709.28	1.96	Oxidation-state component
Used	Fe 2p	Fe(II) $2p_{1/2}$	722.08	1.96	Oxidation-state component
Used	Fe 2p	Fe(III) $2p_{3/2}$	710.81	3.35	Oxidation-state component
Used	Fe 2p	Fe(III) $2p_{1/2}$	723.67	3.33	Oxidation-state component
Used	Fe 2p	Satellite 1 ($2p_{3/2}$ / $2p_{1/2}$)	714.28 / 727.08	4.33 / 4.33	Included in envelope; excluded from state proportions
Used	Fe 2p	Satellite 2 ($2p_{3/2}$ / $2p_{1/2}$)	718.32 / 732.12	4.33 / 4.33	Included in envelope; excluded from state proportions
Used	Co 2p	Co(III) $2p_{3/2}$ / $2p_{1/2}$	779.09 / 794.04	2.38 / 2.38	Oxidation-state components
Used	Co 2p	Co(II) $2p_{3/2}$ / $2p_{1/2}$	780.12 / 795.52	3.33 / 3.33	Oxidation-state components

Sample	Region	Component	Binding energy (eV)	FWHM (eV)	Treatment
Used	Co 2p	Satellite 1 ($2p_{3/2}$ / $2p_{1/2}$)	784.88 / 800.67	3.37 / 3.37	Included in envelope; excluded from state proportions
Used	Co 2p	Satellite 1 ($2p_{3/2}$ / $2p_{1/2}$)	788.47 / 804.20	3.37 / 3.37	Included in envelope; excluded from state proportions

The Shirley background was used.

Table S6. ICP results for FeCo/NPC-800 and the filtrate after the standard 60 min reaction.

Sample	Element	Instrument	Result (mean ± SD)	Unit
Solid FeCo/NPC-800	Fe	ICP-OES	20.638 ± 0.042	wt%
Solid FeCo/NPC-800	Co	ICP-OES	25.449 ± 0.031	wt%
Solid FeCo/NPC-800	Zn	ICP-OES	0.0152 ± 0.0001	wt%
60 min, 0.22 μm filtrate	Fe	ICP-MS	1.632 ± 0.046	mg L^{-1}
60 min, 0.22 μm filtrate	Co	ICP-MS	5.127 ± 0.128	mg L^{-1}
60 min, 0.22 μm filtrate	Zn	ICP-MS	0.0210 ± 0.0010	mg L^{-1}

Table S7. Apparent pseudo-first-order fitting results for Figure 6.

Panel	System/material	Fitting interval	Apparent rate constant (min^{-1})	R^2
6a	PMS	2–30 min	0.0016	0.97
6a	FeCo/NPC-800	2–30 min	0.0142	0.93
6a	FeCo/NPC-800 + PMS	2–30 min	0.06060	0.98
6b	FeCo/NPC-700	2–30 min	0.03034	0.99
6b	FeCo/NPC-800	2–30 min	0.06060	0.98
6b	FeCo/NPC-900	2–30 min	0.05438	0.99
6c	NPC-800	2–30 min	0.000437	0.62
6c	Fe/NPC-800	2–30 min	0.01154	0.82
6c	Co/NPC-800	2–30 min	0.04195	0.84
6c	FeCo/NPC-800	2–30 min	0.06060	0.98

Table S8. Apparent pseudo-first-order fitting results for the operating-condition series in Figure 7.

Variable	Level	Fitting interval	Apparent rate constant (min^{-1})	R^2
Catalyst	0.2 g L^{-1}	2–30 min	0.06060	0.98
Catalyst	0.8 g L^{-1}	2–30 min	0.06602	0.83
Catalyst	1.0 g L^{-1}	2–30 min	0.06574	0.90
pH	2	2–20 min	0.05015	0.81
pH	5	2–20 min	0.06589	0.97
pH	7	2–20 min	0.07121	0.99
pH	9	2–20 min	0.13388	0.98
PMS	0.1 mmol L^{-1}	2–30 min	0.06060	0.98
PMS	0.2 mmol L^{-1}	—	n.f.	—
PMS	0.3 mmol L^{-1}	—	n.f.	—
PMS	0.4 mmol L^{-1}	—	n.f.	—
PMS	0.5 mmol L^{-1}	—	n.f.	—
SDZ	4 mg L−1	—	n.f.	—
SDZ	6 mg L^{-1}	—	n.f.	—
SDZ	8 mg L^{-1}	2–20 min	0.10543	0.99
SDZ	10 mg L^{-1}	2–30 min	0.06060	0.98

n.f., not fitted. A fit was not reported when SDZ fell below the HPLC quantification limit too rapidly to leave at least three quantifiable nonzero concentration points.

Table S9. Preparation-temperature, textural-property, and SDZ-removal comparison.

T (°C)	BET area ($m^2 g^{-1}$)	Total pore volume ($cm^3\ g^{-1}$)	Micropore volume ($cm^3\ g^{-1}$)	Apparent rate constant (min^{-1})	R^2	60 min removal (%)
700	41.59	0.0313	0.0150	0.03034	0.99	67.3
800	423.5	0.3403	0.1522	0.06060	0.98	91.2
900	1289	0.9148	0.4895	0.05438	0.99	93.5

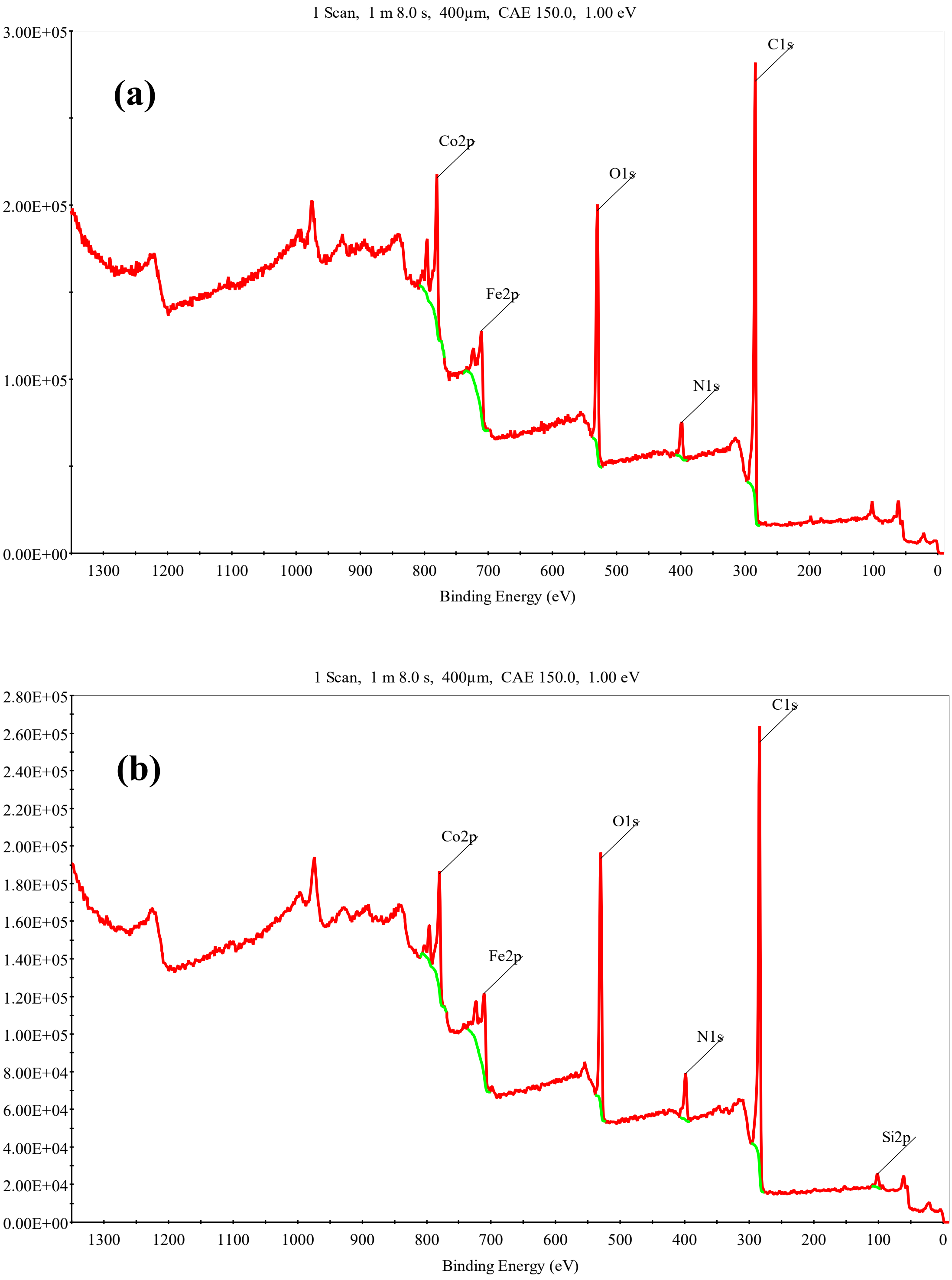

Figure S1. Survey XPS spectrum of FeCo/NPC-800 before reaction (a) and after reaction (b).

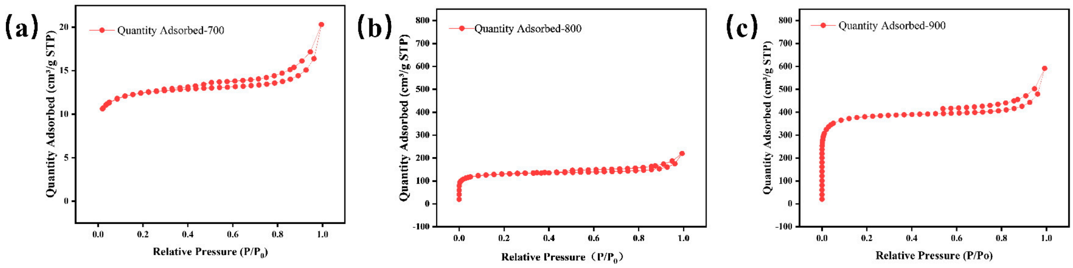

Figure S2. Nitrogen adsorption－desorption isotherms of different FeCo/NPC samples: (a) FeCo/NPC-700; (b) FeCo/NPC-800; (c) FeCo/NPC-900.

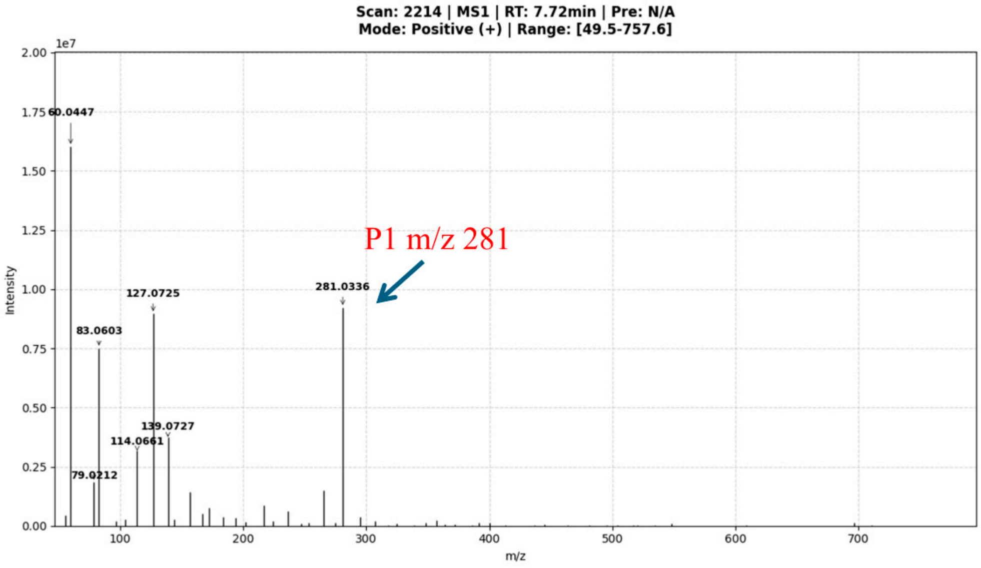
Scan: 2214 | MS1 | RT: 7.72min | Pre: N/A
Mode: Positive (+) | Range: [49.5-757.6]
1e7
Intensity
m/z
60.0447
83.0603
127.0725
114.0661
139.0727
79.0212
281.0336
P1 m/z 281

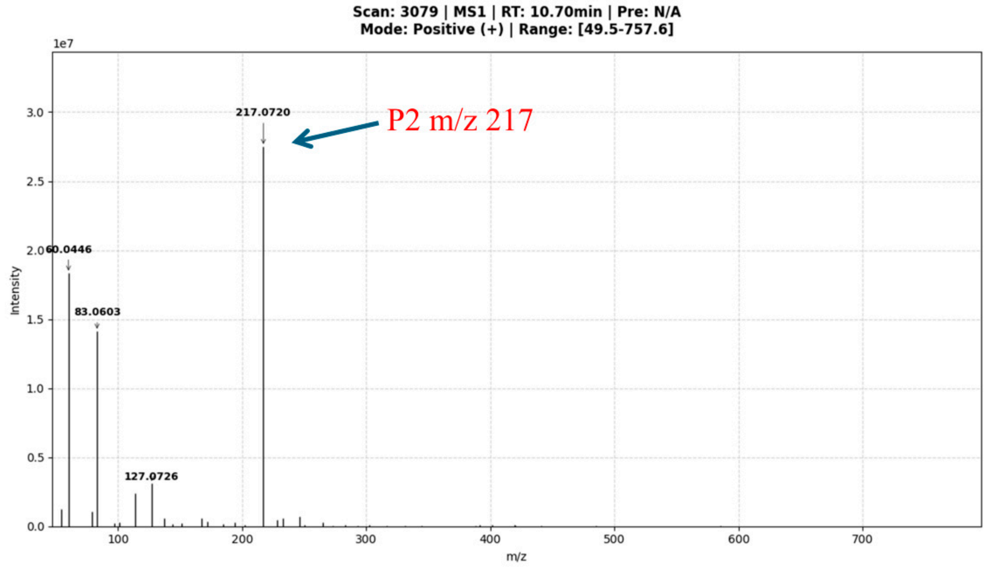
Scan: 3079 | MS1 | RT: 10.70min | Pre: N/A
Mode: Positive (+) | Range: [49.5-757.6]
1e7
Intensity
m/z
217.0720
P2 m/z 217
60.0446
83.0603
127.0726

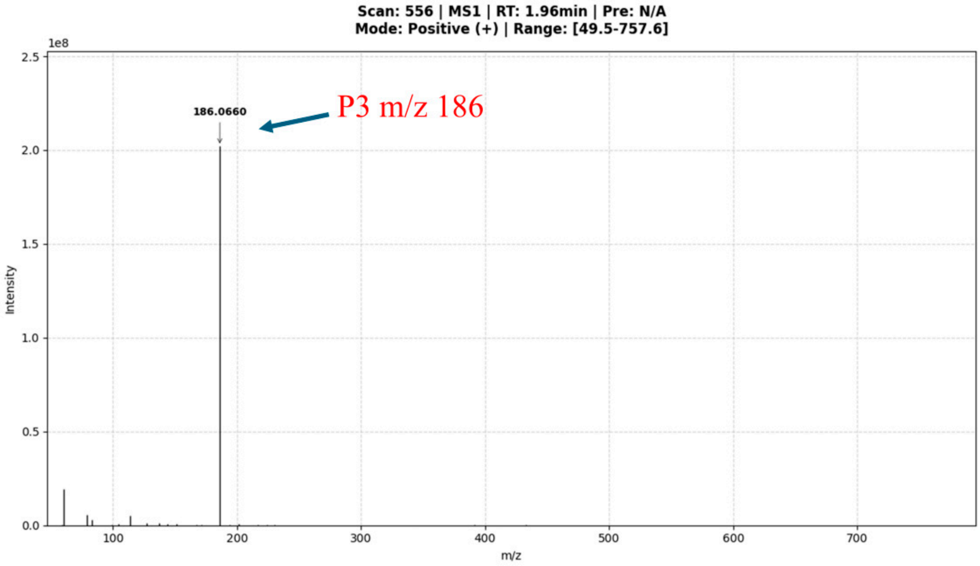
Scan: 556 | MS1 | RT: 1.96min | Pre: N/A
Mode: Positive (+) | Range: [49.5-757.6]
1e8
186.0660
P3 m/z 186
Intensity
m/z

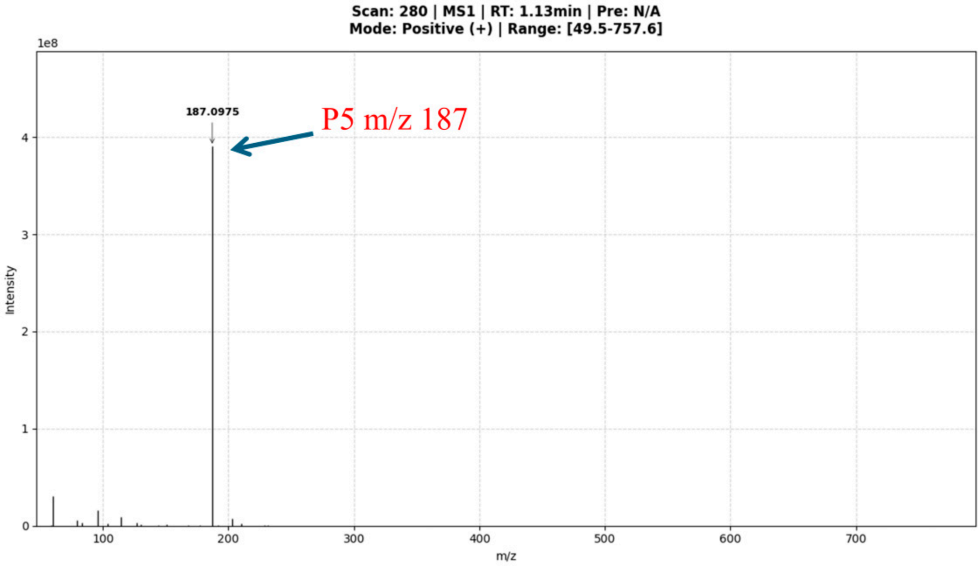
Scan: 280 | MS1 | RT: 1.13min | Pre: N/A
Mode: Positive (+) | Range: [49.5-757.6]
1e8
187.0975
P5 m/z 187
Intensity
m/z

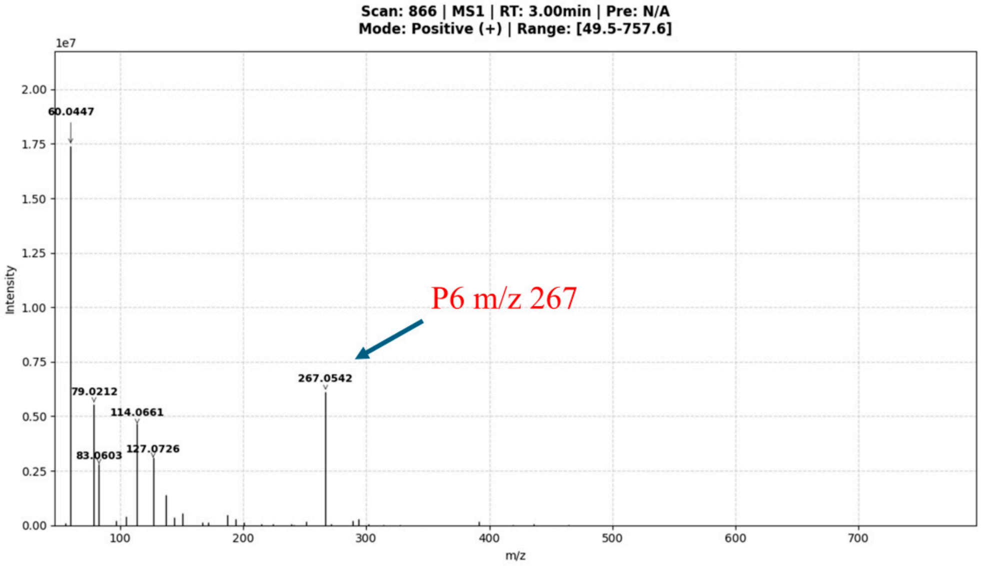
Scan: 866 | MS1 | RT: 3.00min | Pre: N/A
Mode: Positive (+) | Range: [49.5-757.6]
1e7
Intensity
m/z
60.0447
79.0212
83.0603
114.0661
127.0726
267.0542
P6 m/z 267

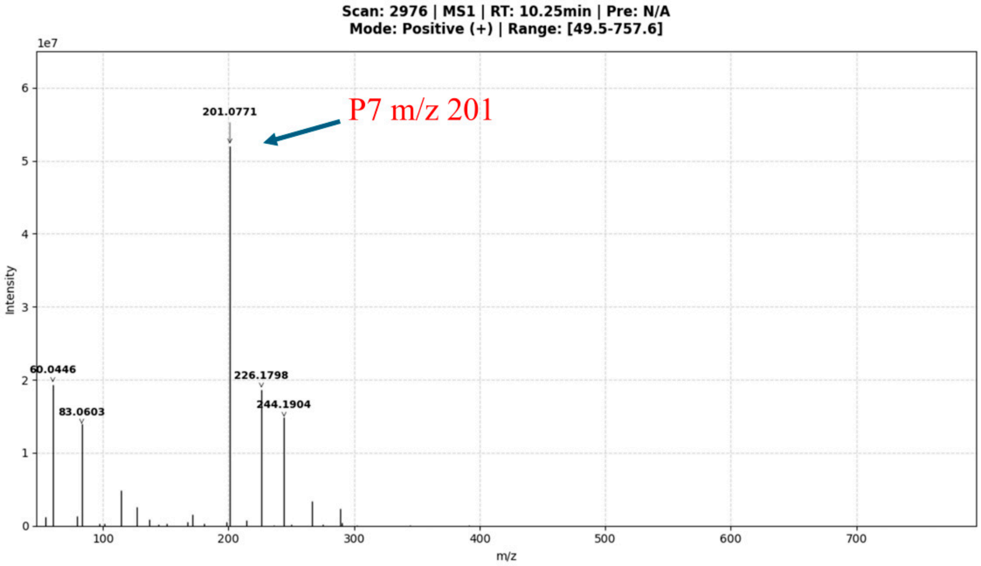
Scan: 2976 | MS1 | RT: 10.25min | Pre: N/A
Mode: Positive (+) | Range: [49.5-757.6]
1e7
Intensity
m/z
201.0771
P7 m/z 201
60.0446
83.0603
226.1798
244.1904

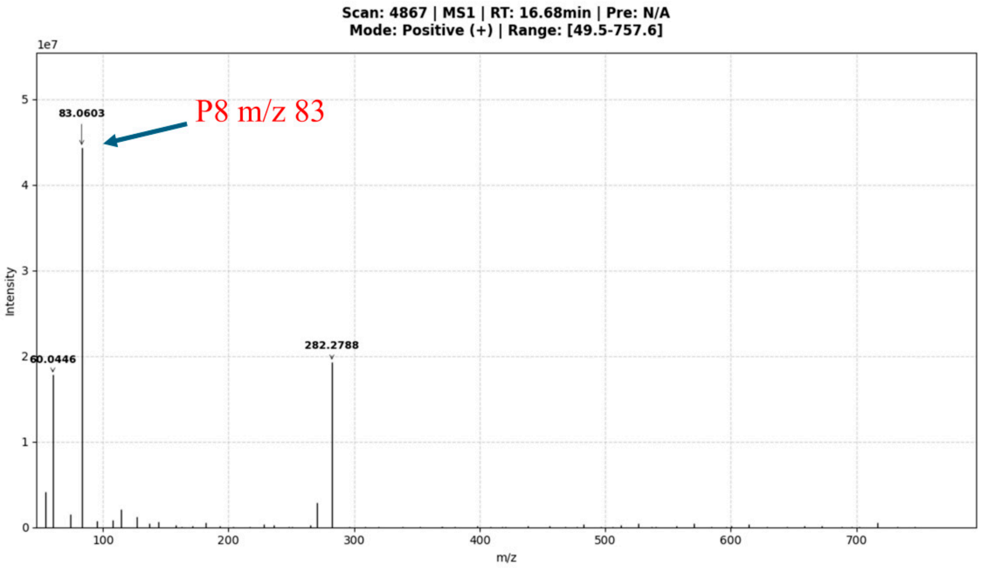

Figure S3. LC-MS spectra with annotated peaks.

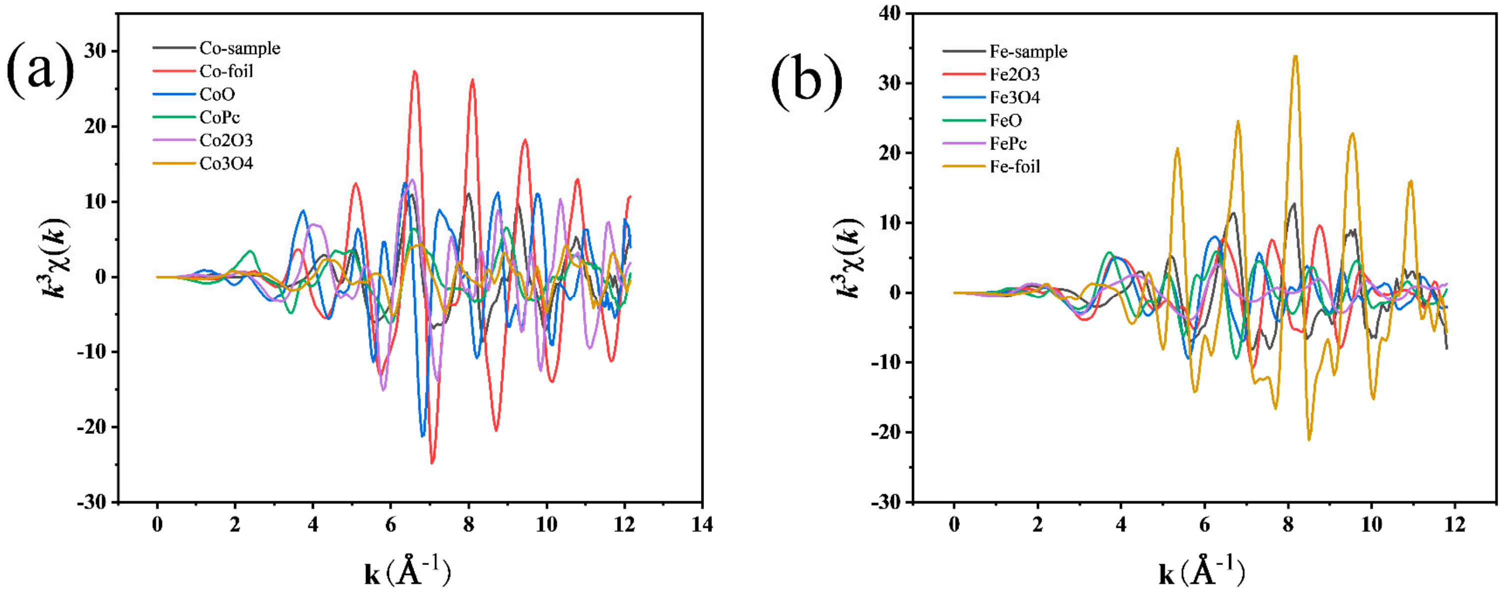

Figure S4. Full-spectrum k-space oscillation waveform of FeCo/NPC-800.

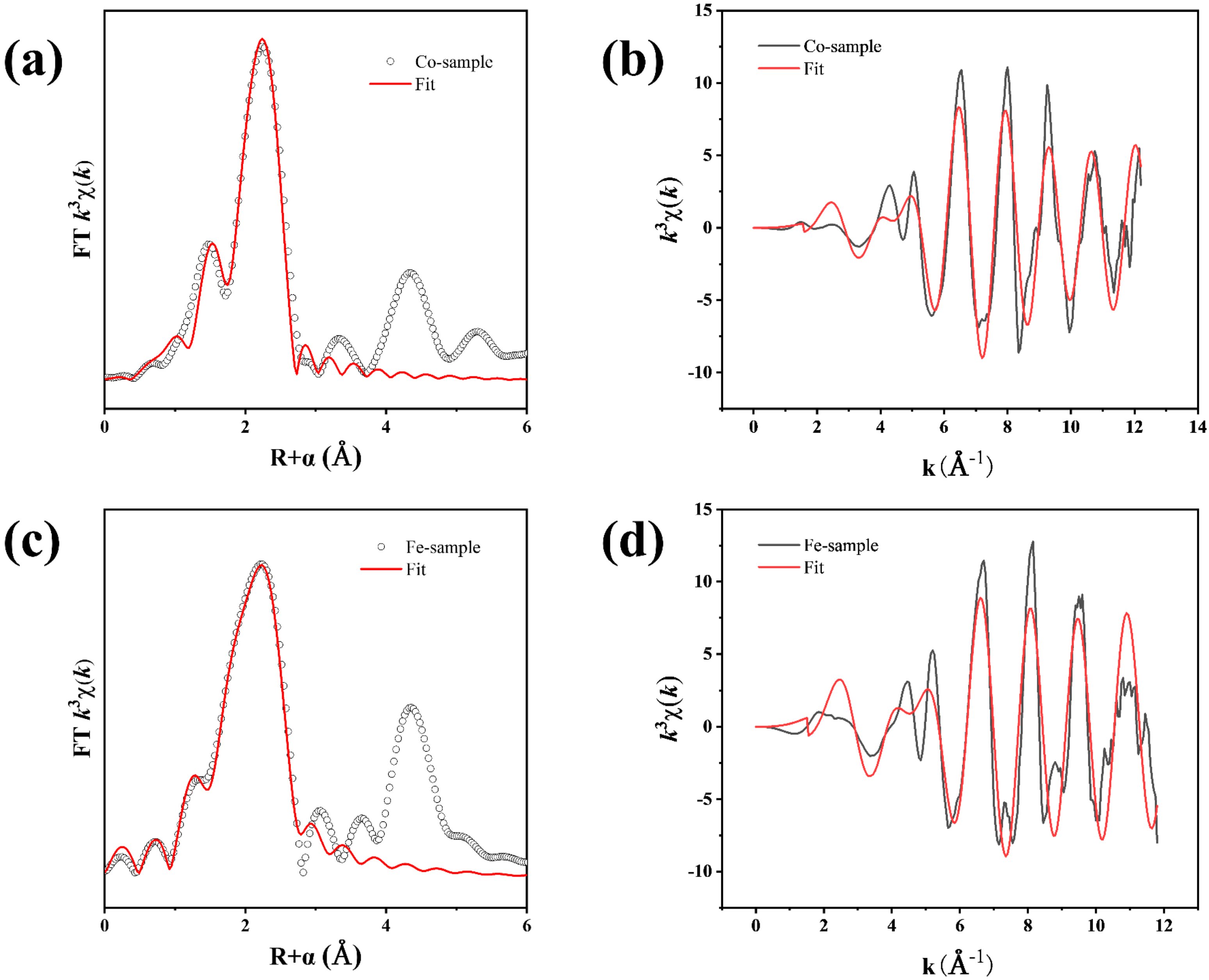

Figure S5. Comparison plot of fitted curves of FeCo/NPC-800.

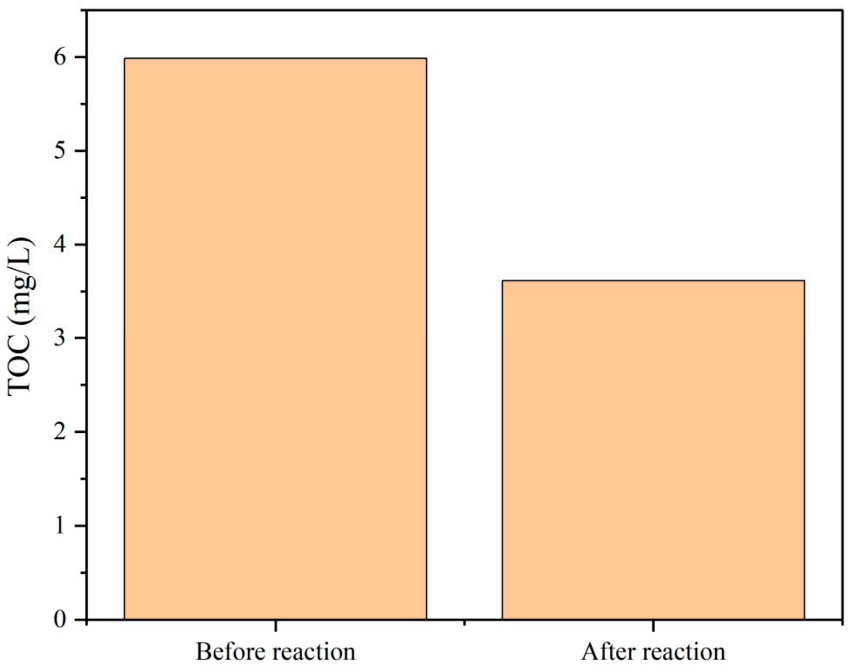

Figure S6. TOC removal before and after catalytic reaction over FeCo/NPC-800.

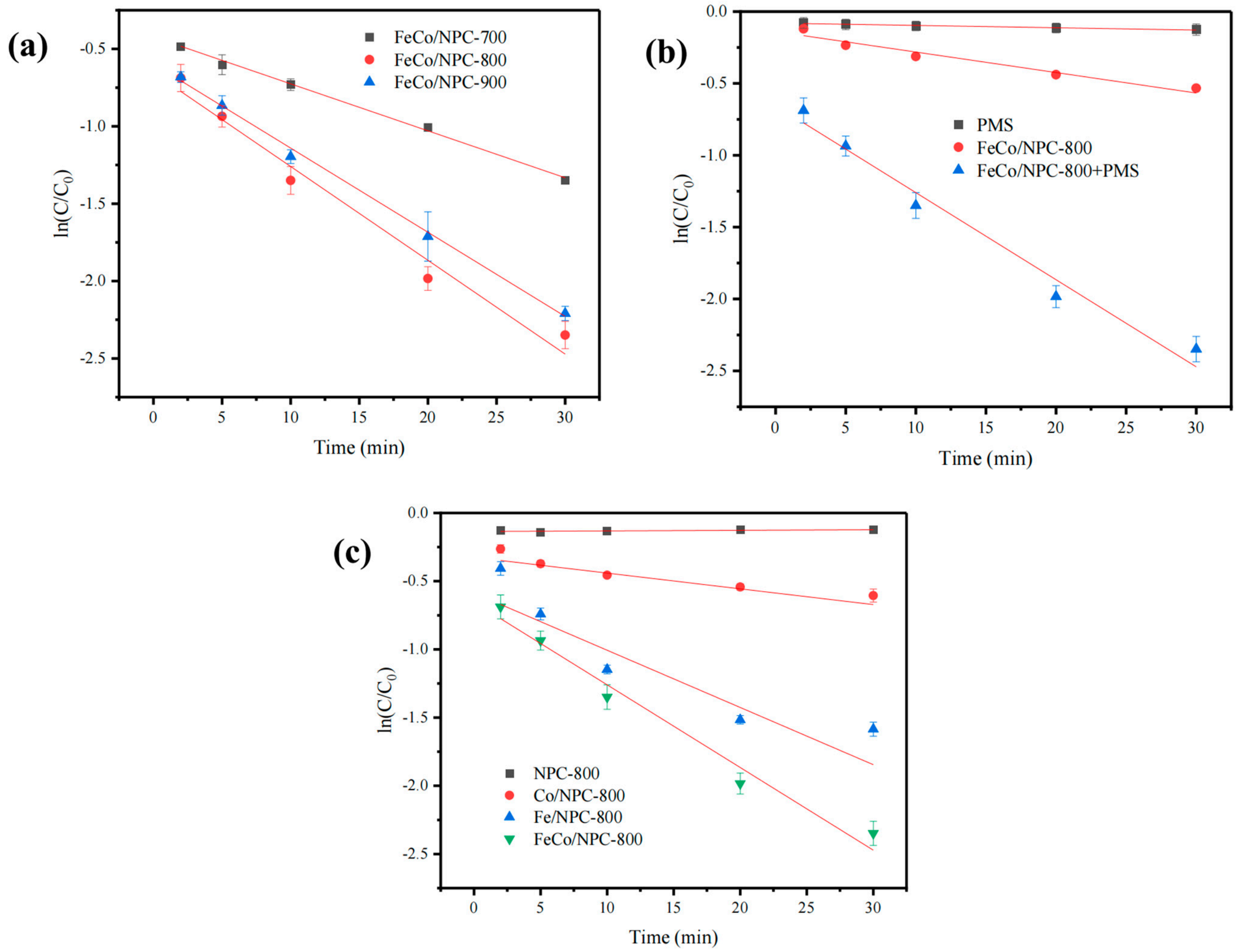

Figure S7. Apparent pseudo-first-order plots over 2–30 min for (a) FeCo/NPC-700, FeCo/NPC-800, and FeCo/NPC-900; (b) PMS alone, FeCo/NPC-800 alone, and FeCo/NPC-800/PMS; and (c) NPC-800, Fe/NPC-800, Co/NPC-800, and FeCo/NPC-800 in the presence of PMS.

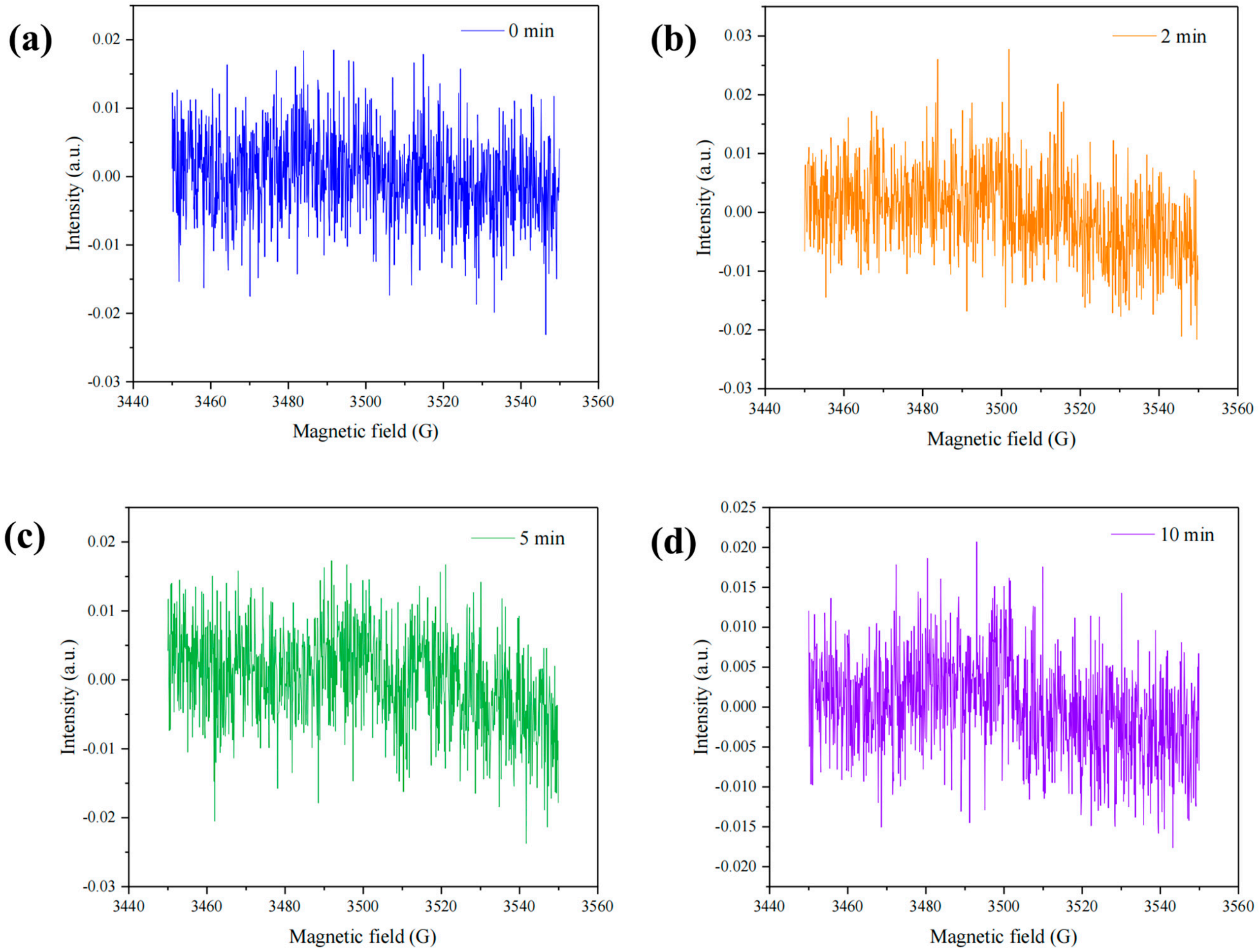

Figure S8. TEMP-EPR spectra of the FeCo/NPC-800/PMS system without SDZ in the dark at 0, 2, 5, and 10 min.
